# Molecular mechanism of synergy between the antimicrobial peptides PGLa and magainin 2

**DOI:** 10.1038/s41598-017-12599-7

**Published:** 2017-10-13

**Authors:** Jonathan Zerweck, Erik Strandberg, Olga Kukharenko, Johannes Reichert, Jochen Bürck, Parvesh Wadhwani, Anne S. Ulrich

**Affiliations:** 1 Karlsruhe Institute of Technology (KIT), Institute of Organic Chemistry, Fritz-Haber-Weg 6, 76131 Karlsruhe, Germany; 2 KIT, Institute of Biological Interfaces (IBG-2), POB 3640, 76021 Karlsruhe, Germany

## Abstract

PGLa and magainin 2 (MAG2) are amphiphilic α-helical membranolytic peptides from frog skin with known synergistic antimicrobial activity. By systematically mutating residues in the two peptides it was possible to identify the ones crucial for the synergy, as monitored by biological assays, fluorescence vesicle leakage, and solid-state ^15^N-NMR. Electrostatic interactions between anionic groups in MAG2 and cationic residues in PGLa enhance synergy but are not necessary for the synergistic effect. Instead, two Gly residues (7 and 11) in a so-called GxxxG motif in PGLa are necessary for synergy. Replacing either of them with Ala or another hydrophobic residue completely abolishes synergy according to all three methods used. The designer-made peptide MSI-103, which has a similar sequence as PGLa, shows no synergy with MAG2, but by introducing two Gly mutations it was possible to make it synergistic. A molecular model is proposed for the functionally active PGLa-MAG2 complex, consisting of a membrane-spanning antiparallel PGLa dimer that is stabilized by intimate Gly-Gly contacts, and where each PGLa monomer is in contact with one MAG2 molecule at its C-terminus.

## Introduction

All organisms are exposed to a large number of microbial species. Innate immune defense systems have emerged as a first line of protection against potential pathogens. Antimicrobial peptides (AMPs), also known as host defense peptides, are found in almost all types of organisms as part of this innate immunity^1–3^. These peptides are often short (10-30 amino acids), cationic, and can form amphipathic structures, and many of them are membrane-active^1–6^. AMPs are supposed to be less likely than traditional antibiotics to induce resistance in bacteria under natural conditions^7,8^, although resistant strains can evolve under intensive selection in the laboratory^9,10^. They promise to be a possible alternative to treat multiresistant bacteria, which pose an increasing threat to human health^7,8,11^. Hence, there is a strong interest in understanding the mechanism of action of AMPs in the membrane.

**Figure 1:**
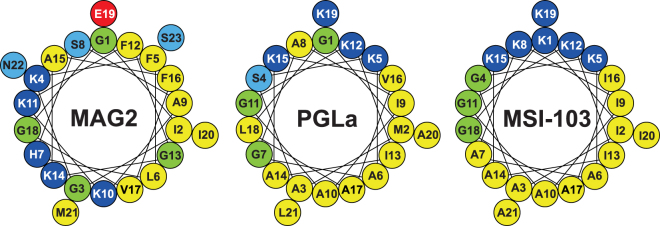
Helical wheels of MAG2, PGLa and MSI-103. In this and later figures, amino acids are color-coded: hydrophobic residues in yellow; cationic in dark blue; anionic in red; polar in light blue; and Gly in green.

A particularly intriguing phenomenon is the synergy between specific AMPs. It has been observed in some cases that the antimicrobial effect of such combination is much higher than for each peptide alone, and using a mix of AMPs may be beneficial and also reduce the risk of resistance evolving^12^. Of special interest is the synergy between peptides that are naturally in contact *in vivo*, as for example reported for two AMPs from human platelets^13^. A well-known synergistic pair of this type is PGLa (GMASKAGAIAGKIAKVALKAL-NH_2_) and magainin 2 (MAG2) (GIGKFLHSAKKFGKAFVGEIMNS-OH), both of which are found in the skin of the African frog *Xenopus laevis* ^14–16^. By circular dichroism spectroscopy (CD) it has been shown that both peptides are unstructured in aqueous solution, but form amphipathic α-helices in membranes^17–19^. Helical wheel projections are shown in Fig. 1. It can be seen that as an α-helix, both peptides have one mainly hydrophobic face and one polar (charged) face.

Since both peptides are simultaneously present at the same site of action, the antimicrobial activity of the frog skin is greatly enhanced by this synergy. The synergistic effect was first noted in studies of membrane potential dissipation^20^, and later demonstrated by the release of carboxyfluorescein from liposomes^21^, membrane permeabilization^22^, antimicrobial and anticancer activities, and liposome permeabilization^23^. The fact that synergy is observed in all these different assays indicates that the effect is based on the primary activity of the peptides, i.e. the permeabilization of membranes. This mode of action has been proposed to involve the formation of water-filled pores that are lined by the membrane-spanning amphiphilic peptides^16,23–27^. Certain mutants of PGLa and MAG2 have also shown synergistic antimicrobial activity^25,28,29^.

In leakage studies, mixtures of PGLa and MAG2 were found to induce maximum permeabilization in different membranes at a 1:1 molar ratio, which led to the hypothesis that the peptides form heterodimers in the membrane^25,30^. The study by Matzusaki *et al*. also showed that the putative heterodimeric complex, as well as each component peptide on its own, can form pores^25^. The rate of pore formation was reported to follow the order of PGLa/MAG2 complex ≥ PGLa > MAG2, whereas lifetime of the pores was in the order MAG2 > PGLa/MAG2 complex > PGLa. Thus, synergy seems to be due to the formation of a heteromolecular complex, characterized by fast pore formation and reasonable pore stability^25^. We have previously observed that the rate of vesicle leakage followed the order PGLa/MAG2 complex ≥ PGLa > MAG2^30^.

In several studies it has been shown that magainin and related peptides can form dimers^25,31–34^, and that dimerization is important for activity. For example, the membrane permeabilization and antimicrobial activity of disulfide-linked MAG2 homodimers was higher than that of the wild type peptide^35,36^. Some heterodimers of AMPs are also known in nature. For example, distinctin is a known AMP from the tree-frog *Phyllomedusa distincta*, and consists of two different polypeptide chains connected by a disulfide bridge^37^. It is thus not surprising that PGLa/MAG2 heterodimers can form and be more active than either peptide alone^25^. In cross-linking experiments it was found that PGLa and MAG2 are preferentially active as parallel heterodimers in PC/PG membranes^38^. The membrane permeabilization activity of these cross-linked parallel heterodimers was considerably higher than for each peptide alone, supporting the hypothesis that synergy is linked to heterodimer formation.

To get a more detailed structural picture of the peptides in membranes, the orientation of PGLa and MAG2 has been studied extensively with solid-state NMR, a well-established method to determine helix orientations in hydrated lipid bilayers under quasi-native conditions^39–43^. In DMPC/DMPG lipids, MAG2 alone was found to lie flat on the membrane surface in a so-called surface state (“S-state”) at peptide-to-lipid ratios between P/L = 1/500 and 1/50^30,44,45^. Only at a very high peptide concentration of 1/10, a more tilted “T-state” was observed^45^. PGLa alone was in an S-state at low concentration, but reoriented to a T-state at P/L = 1/100 or above^30,45–48^. In a 1:1 mixture of the two peptides, PGLa was observed in a transmembrane “inserted state” (“I-state”)^30,45,49,50^, while MAG2 reoriented to the tilted T-state^44,45,50^. From these data, and based on the heterodimer hypothesis mentioned above, it was proposed that PGLa and MAG2 form a complex in the membrane that leads to the formation of a stable water-filled pore^25,44,49^. In these pores, transmembrane PGLa peptides line the walls, while MAG2 stays on the bilayer surface and stabilizes the pore by specific interactions with PGLa^44,45,50^. Any details on the molecular interactions, however, remained completely elusive.

We have recently shown that the membrane-bound PGLa-MAG2 complex is thermodynamically more stable than PGLa-PGLa oligomers, which are in turn more stable than MAG2-MAG2 oligomers^30^. This order was derived from the Hill coefficients (*n*) determined from a vesicle leakage assay; *n* ≈ 2 for MAG2-MAG2, *n* ≈ 4 for PGLa-PGLa, and *n* ≈ 7 for PGLa-MAG2 (1:1 molar mixture). Free energy calculations also gave the same order of interaction strengths, with dimerization energies of around -4 kJ/mol for PGLa/MAG2 heterodimers, -2.4 kcal/mol for PGLa/PGLa and -1.8 kcal/mol for MAG2/MAG2 homodimers^30^. Using another methodology, Matsuzaki *et al*. got similar dimerization free energy values^25^. At peptide-to-lipid ratios below 1:300, no interaction was detected between PGLa and MAG2 in solid-state NMR experiments, and only at higher concentrations a concerted re-orientation of the two peptides was observed^30^. Also, the re-orientation of PGLa in the presence of MAG2 was only seen in lipid systems with a positive spontaneous curvature, like DMPC/DMPG^45^. In contrast, in lipid systems with a negative spontaneous curvature, like POPC/POPG, both peptides always remained in the S-state, either on their own as well as in 1:1 mixtures. Thus, complex formation depends strongly on the membrane environment, and peptide-peptide interactions do not seem to be very strong. Nevertheless, this interaction significantly enhances their antimicrobial activity and vesicle leakage by more than one order of magnitude^30^.

It would now be highly interesting to determine the actual molecular structure of the membrane-bound PGLa-MAG2 complex, about which very little is known. So far, many studies have been performed to improve the antimicrobial activity of MAG2 on its own, by implementing different mutations^51–58^. In particular, it was found that the antimicrobial effect was strongly enhanced by replacing Gly residues with Ala^51,52^, or by increasing the positive charge of MAG2, for example by amidating the C-terminus^52,58^. Also by increasing the hydrophobicity or the hydrophobic moment, the antimicrobial activity could be improved^53–57^. However, the synergy of MAG2 with PGLa has only rarely been investigated, and no clear understanding has emerged^25,28,29^.

In the present study, we set out to pinpoint those key residues in the two peptides that are necessary for their interaction. We synthesized a large number of mutants of the two peptides, and also of another designer-made AMP called MSI-103 ([KIAGKIA]_3_-NH_2_), which had been derived from PGLa^52,59,60^. We then studied the synergy of either PGLa or MSI-103 with MAG2, systematically for all the mutants, using solid-state NMR, vesicle leakage, as well as antimicrobial activity. From these experiments, we could identify key residues that are responsible for the synergy in both peptides, and from which a model of the three-dimensional architecture of the PGLa-MAG2 complex in the active membrane-bound pore could be derived.

## Results and Discussion

### Comparison of methods

Three different methods are used in this work to study synergy between the peptides. At a first glance, it may seem that these three methods, checker-board assays﻿, NMR and leakage, are working under very different conditions, and are not measuring the same thing (Table 1). However, from many previous studies from our group and others during the last 20 years, it is known that synergy between PGLa and MAG2 is observed using all three methods under suitable conditions, which has been established previously^16,25,45,49^. Our assumption is that the synergy is due to peptide-peptide interactions, which are involved in all the different kinds of synergistic activity studied here.

**Table 1:** Methods used to study synergy between PGLa and MAG2 in this study. Approximate values are given.

Method	Measures	Sample volume	Lipid conc.	Peptide conc.	P/L	Lipid	Lipid spont. curvature
Checker-board	Killing of bacteria	100 µL	2 nM	8 µM	4000:1	Bacterial lipids	probably negative
Leakage	Leakage of dye from lipid vesicles	1500 µL	100 µM	0.6 µM or 3.3 µM	1:160 or 1:30	POPC/POPG or POPE/POPG/TOCL	negative
^15^N-SSNMR	Orientation of peptides in membranes	30 µL	800 mM	16 mM	1:50	DMPC/DMPG	positive

A suitable method to study synergistic antimicrobial activity is the so-called checker-board assay, described in the Methods section. We performed such experiments on one Gram-negative bacterium, *E. coli*, and one Gram-positive one, *S. aureus*. From this method a fractional inhibitory concentration index (FICI) ﻿is determined, and when this is below 0.5, the peptides are said to have a synergistic effect against bacteria. This is the biologically relevant effect, but we are here interested in the atomistic details of the interactions behind the effect. Therefore we also use biophysical methods.

The synergy was also investigated by a fluorescence ANTS/DPX leakage assay^24,27,30^. As described in the Methods section, leakage from POPC/POPG (3:1) vesicles was measured for 10 minutes after the addition of vesicles to a peptide solution. Leakage is expressed as percentage of the full leakage, which was obtained after addition of detergent. A synergy factor was defined to describe the enhancement of leakage of the mixture compared to the sum of the extent of leakage for each component. The PGLa-WT/MAG2-WT mixture was measured in each series as a control and usually gave a synergy factor around 11. In general a synergy factor above 2 indicated synergy. The idea behind this method is that the two peptides are active against bacteria by attacking the membranes, forming pores that leads to leakage of the bacterial membranes. In the leakage assay, this pore formation can be studied under more controlled conditions. Here we use two lipid compositions for the vesicles, which are mimicking bacterial membranes.

When a peptide is known to be α-helical in the presence of membranes, the orientation of the helix can be determined by ^15^N-NMR in oriented lipid bilayers, using peptides labeled with ^15^N at one position^42,61,62^. The angle of the helix axis with respect to the membrane normal can be estimated from the ^15^N chemical shift: a chemical shift of around 90 ppm means that the helix axis is perpendicular to the membrane normal (i.e., the peptide is flat on the surface), while a chemical shift around 200 ppm means that the helix axis is parallel to the membrane normal (i.e., the peptide is in a transmembrane orientation). We have previously shown that PGLa on its own in DMPC/DMPG, at a P/L of 1:50, is in a tilted “T-state” with a tilt angle of around 125°^16,46–48^. This corresponds to a ^15^N chemical shift of around 130 ppm. In the presence of MAG2 at a 1:1 molar ratio, PGLa flips into a so-called “inserted state” (I-state) with a transmembrane orientation, which leads to a ^15^N chemical shift of around 200 ppm. This re-orientation of PGLa may explain the synergistic effects between the two peptides, as it agrees with a pore model of action^16,49^. Assuming that the insertion of PGLa is due to specific synergistic interactions with MAG2, we prepared samples with^ 15^N-labeled PGLa-WT and mutants and used^ 15^N-NMR to find out whether the re-orientation still occurred when the unlabeled MAG2 and its mutants were added. The idea behind this kind of experiment is that the pore, which leads to leakage and killing of bacteria, are formed by transmembrane PGLa molecules lining the pore. When peptide-peptide interactions are removed, so that PGLa is not inserting, then pores are not formed, and accordingly there should also be no leakage and no bacterial killing.

**Figure 2:**
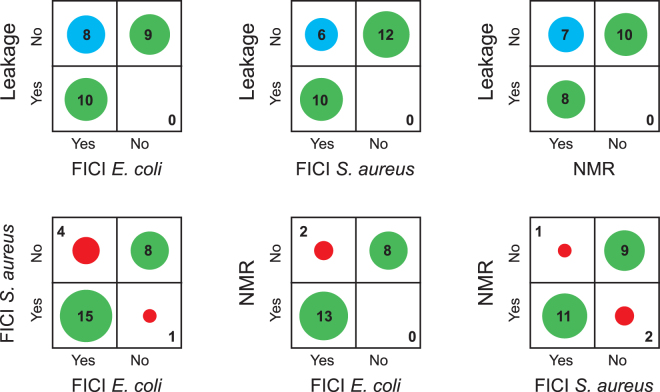
Correlations between results from the three different methods used to determine synergy. Methods are compared pair-wise, and the number of peptide pairs showing synergy or not with the two methods are plotted. Green circles indicate that the two methods gave the same result. In blue are shown cases where leakage did not show synergy but the other method did. In red are shown other cases where the two methods gave different results.

If these key assumptions are wrong, then we can expect that the different methods give different results. On the other hand, if all methods give the same results this is a validation of the model of action of the peptides. As illustrated in Fig. 2, from our large set of mutants of PGLa and MAG2, we see a high correlation between the methods. In most cases (green circles), the three methods give the same result (synergy or no synergy). And in those cases where there is a difference, we note that it is always the case that leakage is not showing synergy whereas the other two methods do show synergy (blue circles), while it never happens that leakage shows synergy but not the other methods. It seems that the leakage assay is the most sensitive to a small reduction in peptide-peptide interactions. Why this is the case is discussed more in detail in the Supplementary Information, where also the correlations are discussed in more depth. We can also note that NMR and FICI are better correlated than leakage and FICI, and that the correlation between FICI in *E. coli* and *S. aureus* is less than between each species and NMR, which might be unexpected, but show that insertion of PGLa in the presence of MAG2 in NMR experiments is a very good predictor of synergy also in bacteria. In short, we conclude that the NMR experiments and also checker-board assays are done under conditions favoring pore formation, but leakage is done under conditions unfavorable to pore formation. In NMR, essentially all peptides are forming a pore, but in leakage only a very small proportion of pores are formed, which is however enough to give vesicle leakage in a few minutes. When now the peptide-peptide interactions are reduced by a mutation in the peptide sequence, a small reduction may be enough to decrease the numbers of pores so that leakage is reduced, but for NMR or FICI, a small reduction in the interaction strength may not be enough to block all peptides from inserting into the membrane.

For the purpose of our study, we can now combine the strengths of the different methods. When we try to find amino acids responsible for synergy, we are doing two kinds of experiments. When we try to remove synergy, we want to find residues that completely abolish it, so that we want all three methods to show no synergy, also the more robust synergy seen in NMR. On the other hand, when we want to find a new synergistic peptide, we want all three methods to show synergy, even the most sensitive synergy seen in leakage.

### Electrostatic interactions

**Figure 3:**
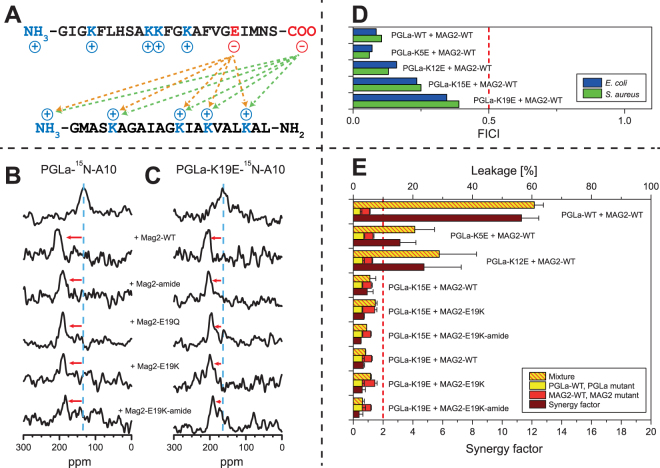
Synergy results for PGLa charge mutants. (**A**) Potential electrostatic interactions between MAG2 (top) and PGLa (bottom). (**B**) ^15^N-NMR spectra of ^ 15^N-labeled PGLa, alone or in 1:1 mixtures with MAG2 and its charge mutants. PGLa on its own gives a chemical shift of around 130 ppm (blue dashed line), which corresponds to a tilted T-state, but in combination with MAG2-WT gives a shift of around 200 ppm, corresponding to an inserted I-state. In the presence of charge mutants of MAG2, the signal of PGLa moves close to 200 ppm in all cases, indicating that the peptide always remains in the I-state. (**C**) ^15^N-NMR spectra of ^15^N-labeled PGLa-K19E, alone or in 1:1 mixtures with MAG2 and its charge mutants. PGLa-K19E on its own gives a chemical shift of around 160 ppm (blue dashed line), but in combination with MAG2-WT or charge mutants thereof, the signal of PGLa-K19E moves close to 200 ppm in all cases, indicating that the peptide always remains in the I-state. (**D**) FICIs determined from combinations of PGLa charge mutants and MAG2-WT. All FICIs are below 0.5 (red dashed line), which indicates synergistic activity. (**E**) Leakage of PGLa charge mutants combined with MAG2-WT and MAG2 mutants in POPC/POPG (3:1) vesicles. For each combination, the leakage was measured for each peptide alone (red and yellow bars) and in 1:1 mixtures (red-yellow striped bars). The synergy factor (brown bars) is the enhancement of leakage of the mixture compared to the sum of the leakage of the single components. Synergy factors above 2 (red dashed line) indicate synergistic activity. All peptide combinations that showed synergy are marked with a red arrow in the NMR spectra.

Electrostatic interactions such as salt bridges between anionic and cationic residues in membrane-bound peptides and proteins have been shown to play a role in the assembly of oligomers^63–68^. PGLa is a cationic peptide with four Lys and a charged N-terminus, while MAG2 has four Lys, one His, one Glu, and is charged at both the N- and the C-terminus (Fig. 3A). Negative charges are not present in PGLa, but in MAG2 there are negative charges at E19 and at the C-terminus, hence numerous interactions are conceivable from these positions with any of the positive charges on PGLa, as shown in Fig. 3A.

To investigate the relevance of these potential electrostatic interactions, we designed 9 different peptide mutants (see Table 2) in which charged residues were removed or exchanged to oppositely charged amino acids. In MAG2, the C-terminus was amidated to remove the anionic group at the terminus, and E19 was replaced with Q or K to either remove this negative charge or even replace it with a positive one. Both negative charges on MAG2 were removed at the same time in the amidated E19K mutant. In PGLa, the Lys residues were exchanged with Glu, one at a time. Here, the idea was both, to block possible salt bridges, and to possibly restore them by combinations with the MAG2-E19K mutants.

**Table 2:** Peptides with charge mutations used to study electrostatic interactions. Mutations of the wild type peptides are marked in bold and ^15^N-labeled residues in bold underline.

Peptide	^15^N-label	Sequence
MAG2-WT	—	GIGKFLHSAKKFGKAFVGEIMNS-OH
MAG2-amide	—	GIGKFLHSAKKFGKAFVGEIMNS-**NH**_**2**_
MAG2-E19Q	—	GIGKFLHSAKKFGKAFVG**Q**IMNS-OH
MAG2-E19K	—	GIGKFLHSAKKFGKAFVG**K**IMNS-OH
MAG2-E19K-amide	—	GIGKFLHSAKKFGKAFVG**K**IMNS-**NH**_**2**_
PGLa-^15^N-A10	Ala10	GMASKAGAI**A**GKIAKVALKAL-NH_2_
PGLa-K5E	—	GMAS**E**AGAIAGKIAKVALKAL-NH_2_
PGLa-K12E	—	GMASKAGAIAG**E**IAKVALKAL-NH_2_
PGLa-K15E	—	GMASKAGAIAGKIA**E**VALKAL-NH_2_
PGLa-K19E	—	GMASKAGAIAGKIAKVAL**E**AL-NH_2_
PGLa-K19E-^15^N-A10	Ala10	GMASKAGAI**A**GKIAKVAL**E**AL-NH_2_

All peptides were studied by CD to check whether the mutations may have changed the secondary structure. All mutants showed the same CD spectra as the wild type peptides, both in phosphate buffer where they all were unordered, (Supplementary Fig. S1), and in DMPC/DMPG vesicles where all were α-helical (Supplementary Fig. S2).

Here, PGLa and the mutant PGLa-K19E were labeled with ^15^N at the backbone amide of Ala10. As seen in Fig. 3B, PGLa-WT can flip into the I-state in the presence of all charge mutants of MAG2. In the presence of MAG2-amide, MAG2-E19Q, MAG2-E19K, and even MAG2-E19K-amide in which no negative charges are present, the chemical shift of PGLa is always near 200 ppm. Exact ppm values are given in Supplementary Table S1.

Also PGLa-K19E can insert into the membrane, in the presence of all MAG2 charge mutants, as seen in Fig. 3C (chemical shifts are given in Supplementary Table S1). We can thus conclude that charge interactions are not responsible for the interactions between PGLa and MAG2 involved in the upright membrane insertion of PGLa in the presence of MAG2.

**Figure 4:**
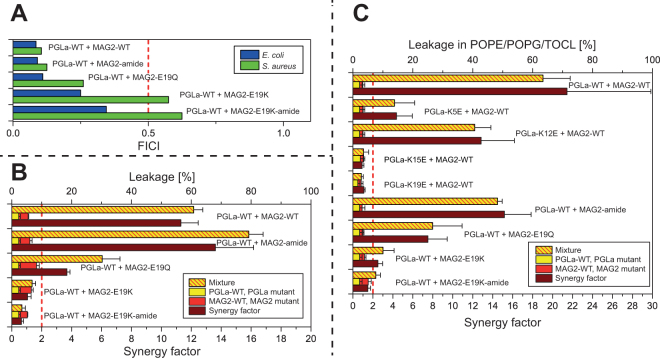
Synergy results for MAG2 charge mutants. (**A**) FICIs determined from combinations of PGLa-WT and charge mutants of MAG2. All peptides show synergy in *E. coli*, but MAG2-E19K and MAG2-E19K-amide are not synergistic in *S. aureus*. (**B**) Leakage of MAG2 charge mutants, alone and in combination with PGLa-WT, in POPC/POPG (3:1) vesicles. (**C**) Leakage of MAG2 and PGLa charge mutants, alone and in combination with PGLa-WT or MAG2-WT, respectively, in POPE/POPG/TOCL (72:23:5) vesicles. The same synergy behavior is seen here as in POPC/POPG (1:1) vesicles.

We performed checker-board assays for all combinations of PGLa and MAG2 and their mutants, using *E. coli* and *S. aureus* bacteria. All results are illustrated here as bar diagrams, and the exact numbers are summarized in Supplementary Table S2. In Fig. 3D, FICIs are given for the charge mutations of PGLa in combination with MAG2-WT, showing that all of the mutants are still synergistic. However, the FICIs increase slightly along the sequence of PGLa, with mutations closer to the C-terminus having larger values, indicating a possible weak interaction for K15 or K19. FICIs for the charge mutants of MAG2 in combination with PGLa-WT are given in Fig. 4A, and here all mutants show synergy against *E. coli*, while MAG2-E19K and MAG2-E19K-amide have FICIs just above 0.5 against *S. aureus*, which indicates that synergy is slightly reduced in a species-dependent manner. No mutation, however, abolishes the interaction in both species, suggesting that the charge interactions are not crucial for the synergy against bacteria, but may contribute to the stability of the complex.

Leakage experiments were performed in POPC/POPG, and values are given in Supplementary Table S3 and are also presented in bar diagrams in Figs 3E and 4B. As seen in Fig. 3E, PGLa-WT and MAG2-WT showed a strong synergy with a high synergy factor of 11. PGLa-K5E and PGLa-K12E also showed synergy with MAG2-WT, but PGLa-K15E and PGLa-K19E did not show synergy with MAG2-WT. To check whether synergy could be restored by charge replacements, PGLa-K15E and PGLa-K19E were also combined with MAG2-E19K and MAG2-E19K-amide, but did not show synergy either.

Figure 4B shows results of leakage measurements in POPC/POPG of MAG2 charge mutations alone and in combination with PGLa-WT. Here, synergy was found for MAG2-amide and MAG2-E19Q, but not for MAG2-E19K and MAG2-E19K-amide.

It could be argued that POPC/POPG (3:1) is not the most appropriate model system to describe a bacterial membrane, which rarely contains PC lipids. We therefore also performed leakage in another lipid system, POPE/POPG/TOCL (72:23:5), which is a good model of *E. coli* membranes^45,69,70^. This lipid system is less susceptible to leakage than POPC/POPG, hence the experiments were performed with P/L ≈ 1:30, which gave a similar extent of leakage as P/L = 1:160 in POPC/POPG, and with this sample composition it was possible to get a second, independent set of synergy factors. Figure 4C shows these leakage data for PGLa and MAG2 charge mutants in POPE/POPG/TOCL as bar diagrams. It can be seen that the result is almost the same as in POPC/POPG: PGLa-WT/MAG2-WT shows strong synergy, PGLa-K5E and PGLa-K12E also show synergy with MAG2-WT, but PGLa-K15E and PGLa-K19E do not; and MAG2-amide and MAG2-E19Q also show synergy with PGLa-WT, while MAG2-E19K-amide does not. The only exception is MAG2-E19K, which shows synergy in POPE/POPG/TOCL but not in POPC/POPG; this mutant also shows synergy in the checkerboard assay against *S. aureus* but not against *E. coli*. For all of the other mutants discussed below, leakage was also studied both in POPC/POPG as well as POPE/POPG/TOCL, but since the results (synergy or no synergy) were in all cases the same, the latter data will not be presented in the text. All leakage data in POPE/POPG/TOCL (72:23:5) for charge mutants are summarized in Supplementary Table S4.

The leakage results (Fig. 4B,C) show that MAG2-amide and MAG2-E19Q, both of which carry only one negative charge (at Glu19 or the C-terminus, respectively), exhibit synergy with PGLa, whereas MAG2-E19K and MAG2-E19K-amide do not show leakage synergy. This indicates that a single negative charge close to the C-terminus of MAG2 is enough for leakage synergy, but when both charges are removed, synergy is lost. When Glu19 was replaced with Lys, it is conceivable that an intermolecular electrostatic interaction between Lys and the charged C-terminus blocked the interaction with charges on PGLa.

From the perspective of PGLa, both K5E and K12E are synergistic, while K15E and K19E are not. This indicates that positive charges close to the C-terminus of PGLa are important. The fact that both K15E and K19E disturb the leakage synergy might be taken as an indication that both these residues are involved at the same time, possibly via two salt bridges to E19 and to the C-terminus of MAG2. On this assumption, that either K15 or K19 of PGLa forms a salt bridge to E19 of MAG2, one would expect that this salt bridge should also be formed within a pair of peptides whose charges are interchanged, namely by combining PGLa-K15E or PGLa-K19E with MAG2-E19K. However, this scenario was not observed, as synergy was not found for these peptide combinations.

**Figure 5:**
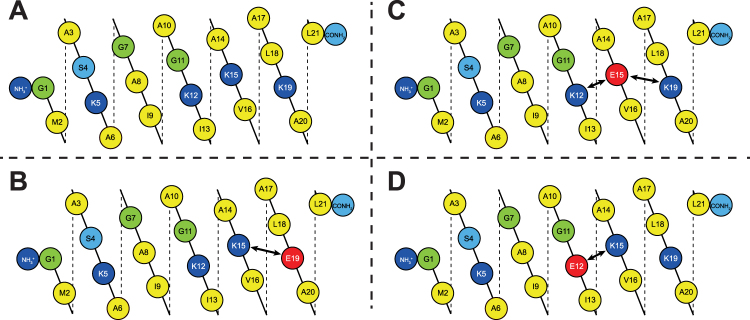
Helical mesh representation of PGLa charge mutants, showing possible intramolecular electrostatic interactions. (**A**) PGLa-WT. (**B**) In PGLa-K19E, the mutation was designed to prevent possible charge interactions between K19 and anionic groups on MAG2, but it could also form a salt bridge to K15 and block this residue from interaction with MAG2. (**C**) Similarly, the PGLa-K15E mutant was designed to prevent possible charge interactions between K15 and MAG2, but a salt bridge to K19 or K12 could block these two residues from interactions with MAG2. (**D**) Likewise, in PGLa-K12E a salt bridge could block K15 from interacting with MAG2.

Another aspect to consider could be intramolecular charge interactions in PGLa within the K → E mutants. Such possibilities are illustrated in Fig. 5. Glu19 of PGLa-K19E could form an intramolecular salt bridge with Lys15, Glu15 of PGLa-K15E with Lys19 or Lys12, and Glu12 of PGLa-K12E with Lys15. In PGLa-K19E and PGLa-K15E, the negative charges on Glu could thereby both block Lys19 and Lys15, and both mutants are indeed no longer synergistic. On the other hand, Glu12 in PGLa-K12E could also block Lys15, but this mutant is still synergistic.

Given that Glu12 in PGLa-K12E cannot block Lys19, we may conclude that probably Lys19 in PGLa is the residue involved in some charge interaction with Glu19 or the C-terminus of MAG2. This would mean that the C-terminus of PGLa is in contact with the C-terminus of MAG2 in the synergistic complex, which is in line with previous results of a cross-linking study in membranes^38^.

To summarize this section, we observe in the leakage assay that charge interactions are involved in leakage synergy, and we can conclude that there are interactions between positive charges in PGLa and negative charges in MAG2 which stabilizes the complex and improves synergy. But on the other hand, NMR still showed some synergy even in the mutant MAG2-E19K-amide, and also the checker-board assay showed synergy for MAG2-E19K-amide (albeit weaker than for MAG2-WT). Therefore, the charge interaction is probably not directly responsible for synergy. It rather seems to stabilize the complex and make the synergy stronger, but even without this interaction, synergy is observed. Hence, there must also be some other interactions present between the two peptides, to be investigated in the next section.

### Sequence comparison between PGLa and MSI-103

**Figure 6:**
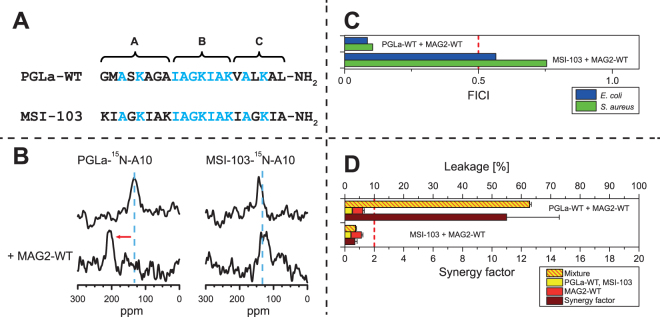
Comparison of synergy with MAG2 for PGLa and MSI-103. (**A)** Comparison of the sequences of PGLa and MSI-103. Identical residues are marked in blue. The central part of both peptides b is identical, but in the N-terminal region a and the C-terminal region c some residues differ. (**B**) ^15^N-NMR spectra of ^15^N-labeled PGLa or MSI-103, alone or in 1:1 mixtures with MAG2-WT. PGLa on its own shows a T-state, and in combination with MAG2-WT an I-state is seen. MSI-103 shows the same T-state with and without MAG2-WT, indicating the absence of synergy. (**C**) FICIs determined from combinations of PGLa or MSI-103 with MAG2-WT. The FICI for PGLa/MAG2 is below 0.5 (dotted line), which indicates synergistic activity, while MSI-103 does not show synergy with MAG2. (**D**) Leakage of PGLa or MSI-103, alone and in combination with MAG2-WT, in POPC/POPG (3:1) vesicles. While PGLa gives a strong synergy, MSI-103 shows only an additive effect in combination with MAG2.

MSI-103 is a designer-made AMP, based on the sequence of PGLa^52^. It has a stronger antimicrobial effect than PGLa, but causes also more side-effects like hemolysis and lipid vesicle fusion^45,59,71,72^. This peptide was not designed to engage in synergy with MAG2, but we wondered whether it could still have a sufficiently similar sequence to PGLa to show such synergy. The helical wheels of PGLa and MSI-103 illustrate the overall similarity in amphipathic structure (Fig. 1). In Fig. 6A the sequences of PGLa and MSI-103 are compared, and 11 out of 21 amino acids which are identical in both peptides are shown in blue. In particular, we can note that all positive charges present in PGLa are also present at the same positions in MSI-103. However, in spite of these remarkable similarities, MSI-103 does not exhibit any synergy with MAG2, as seen from ^15^N-NMR (Fig. 6B), FICIs (Fig. 6C), and leakage (Fig. 6D). This fact opens the possibility to examine the differences between PGLa and MSI-103 as a way of identifying residues involved in the synergistic interaction, which is the subject of the next section.

### From PGLa to MSI-103

The fact that PGLa acts synergistically with MAG2, while MSI-103 does not, suggests that some residue(s) that differs between the two sequences must be responsible for the specific interaction with MAG2. In particular, MSI-103 has all the charges that are present in PGLa at the same positions, but is not synergistic, which means that even if we had shown in Section 2.1 that charge interactions had some small effect, they are not enough to give synergy. One way of identifying the key residues is to mutate residues in PGLa to the analogous ones of MSI-103, and to determine which of these mutations destroy the synergy. To minimize the number of conceivable mutations, a stepwise approach was used. The sequences of all mutants investigated in this section are given in Table 3. CD showed that all peptides were unstructured in solution, and that they formed an α-helix in DMPC/DMPG vesicles, like MSI-103 itself (Supplementary Figs S3 and S4). Supplementary Tables S5–S8 list other data presented in the figures below.

**Table 3:** Peptides used to make PGLa more similar to MSI-103. Mutations leading away from PGLa-WT are marked in bold, and ^15^N-labeled residues in bold underline.

Peptide	^15^N-label	Sequence
PGLa-^15^N-A10	Ala10	GMASKAGAI**A**GKIAKVALKAL-NH_2_
PtM-c1-^15^N-A10	Ala10	GMASKAGAI**A**GKIAK**I**A**G**K**IA**-NH_2_
PtM-a1-^15^N-A10	Ala10	**KI**A**G**K**IAK**I**A**GKIAKVALKAL-NH_2_
PtM-a2-^15^N-A10	Ala10	**KI**A**G**KAGAI**A**GKIAKVALKAL-NH_2_
PtM-a3-^15^N-A10	Ala10	GMASK**IAK**I**A**GKIAKVALKAL-NH_2_
PtM-A6I-^15^N-A10	Ala10	GMASK**I**GAI**A**GKIAKVALKAL-NH_2_
PtM-G7A-^15^N-A10	Ala10	GMASKA**A**AI**A**GKIAKVALKAL-NH_2_
PtM-A8K-^15^N-A10	Ala10	GMASKAG**K**I**A**GKIAKVALKAL-NH_2_
MSI-103-^15^N-A10	Ala10	KIAGKIAKI**A**GKIAKIAGKIA-NH_2_

**Figure 7:**
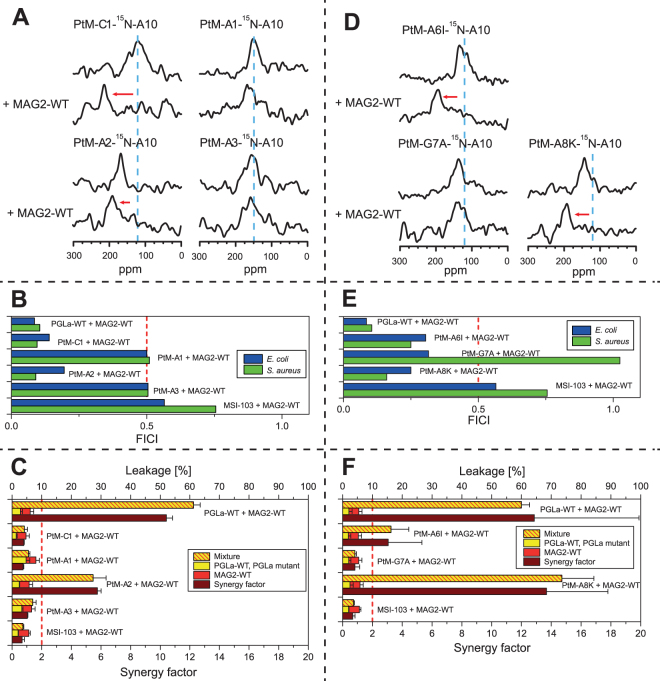
Synergy results for PGLa-to-MSI mutants. (**A**) ^15^N-NMR spectra of ^15^N-labeled PtM mutants, alone or in 1:1 mixtures with MAG2-WT. PtM-c1 and PtM-a2 show synergy, but not PtM-a1 and PtM-a3. (**B**) FICIs determined from combinations of PGLa, PtM mutants, or MSI-103 with MAG2-WT. FICIs below 0.5 (dotted line), indicating synergistic activity, are found for PtM-C1 and PtM-a2, while PtM-a1 and PtM-a3 do not show synergy with MAG2. (**C**) Leakage of PGLa, PtM mutants, or MSI-103, alone and in combination with MAG2-WT, in POPC/POPG (3:1) vesicles. Of the PtM mutants, only PtM-a2 gives a strong synergy. (**D**) ^15^N-NMR spectra of ^15^N-labeled PtM single mutants, alone or in 1:1 mixtures with MAG2-WT. PtM-A6I and PtM-A8K (like PtM-c1) show synergy, but not PtM-G7A. (**E**) FICIs determined from combinations of PtM single mutants with MAG2-WT. FICIs below 0.5 (dotted line), indicating synergistic activity, are found for all peptides in *E. coli*, while in *S. aureus* only PtM-A6I and PtM-A8K show synergy (like PtM-c1), but not PtM-G7A. (**F**) Leakage of PtM single mutants, alone or in 1:1 mixtures with MAG2-WT. PtM-A6I and PtM-A8K show synergy (unlike PtM-c1), but not PtM-G7A.

As seen in Fig. 6A, PGLa and MSI-103 have the same number of residues and can be split into three segments a-b-c of similar size, where the middle segment is identical. In a first step, a peptide was synthesized where segment a in PGLa was replaced with its counterpart in MSI-103. This mutant was called PtM-a1 (“PGLa to MSI-103”, mutation 1 in segment a). Similarly, a mutant was made where segment c was replaced, called PtM-c1. Since the charge mutant study above had indicated some weak interactions involving residues close to the C-termini of both PGLa and MAG2, it could be expected that PtM-c1 would not show synergy. However, both NMR (Fig. 7A) and the FICI (Fig. 7B) indicate that PtM-c1 does engage in synergy with MAG2-WT. Only the more sensitive leakage assay (Fig. 7C) did not show synergy between PtM-c1 and MAG2-WT. Thus, there may be some weak interaction between residues in region c in PGLa and MAG2, but this is not necessary for synergy according to NMR and FICI. On the other hand, PtM-a1 did not show synergy according to NMR (Fig. 7A), FICI (Fig. 7B), or leakage (Fig. 7C). Thus, there must be some strong, specific interaction present in this region, involving at least one of the six amino acids that had been mutated leading away from PGLa-WT.

Two further mutants were prepared next, called PtM-a2 and PtM-a3, in each case converting only three amino acids on PGLa at a time into their counterparts from MSI-103 (Table 3). All our methods, NMR (Fig. 7A), FICI (Fig. 7B), and leakage (Fig. 7C), showed synergy for PtM-a2, but not for PtM-a3. The relevant residues could thus be narrowed down to three possibilities, namely Ala6, Gly7, and/or Ala8 on PGLa. In the next round, three single mutants were thus synthesized: PtM-A6I, PtM-G7A, and PtM-A8K. NMR (Fig. 7D) and leakage (Fig. 7F) showed synergy for PtM-A6I and PtM-A8K, while it was lost for PtM-G7A, and the same result was found in the checker-board assay (Fig. 7E) using *S. aureus*, whereas in *E. coli*, all peptides retained synergy.

To conclude this important section, we have identified Gly7 in PGLa as a crucial residue for its synergy with MAG2. All other changes in the amino acid sequence between PGLa and MSI-103 retained their synergistic interaction according to ^15^N-NMR, meaning none of the other nine differences in the sequence were critical for synergy and still allowed PGLa to re-orient in the presence of MAG2. Also the checker-board assay showed synergy for the other mutants. Only the leakage assay, which is more sensitive than the other two methods towards subtle changes in the interaction, showed some additional effect in the C-terminal region of the peptide. From these results we can conclude that the synergy strongly depends on only one or a few critical residues in PGLa, but apart from this, synergy seems to be quite robust and does not involve extended parts of the sequence. This finding opens the possibility to convert MSI-103 into a synergistic partner of MAG2 by making just a few mutations, which is the subject of the next section.

### From MSI-103 to PGLa

It was possible to abolish the synergy between PGLa and MAG2 with the single mutation of Gly7 to Ala, while all other tested mutations that made PGLa more similar to the non-synergistic MSI-103 did not destroy the synergy. Therefore, it should also be possible to make MSI-103 synergistic by carrying out the opposite mutation of Ala7 to Gly and possibly some additional mutations. Another series of peptides was therefore synthesized, called MtP (“MSI-103 to PGLa”), as listed in Table 4. CD showed that all peptides had a random coil structure in aqueous solution, and formed an α-helix in DMPC/DMPG vesicles, like MSI-103 itself (Supplementary Fig. S5). Supplementary Tables S9–S12 list other data presented in the figures below.

**Table 4:** Peptides used to make MSI-103 more similar to PGLa. Mutations leading away from MSI-103 are marked in bold, and ^15^N-labeled residues in bold underline.

Peptide	^15^N-label	Sequence
MSI-103-^15^N-A10	Ala10	KIAGKIAKI**A**GKIAKIAGKIA-NH_2_
MtP-I6A-^15^N-A10	Ala10	KIAGK**A**AKI**A**GKIAKIAGKIA-NH_2_
MtP-A7G-^15^N-A10	Ala10	KIAGKI**G**KI**A**GKIAKIAGKIA-NH_2_
MtP-K8A-^15^N-A10	Ala10	KIAGKIA**A**I**A**GKIAKIAGKIA-NH_2_
MtP-G4S-A7G-^15^N-A10	Ala10	KIA**S**KI**G**KI**A**GKIAKIAGKIA-NH_2_
MtP-I6A-A7G-^15^N-A10	Ala10	KIAGK**AG**KI**A**GKIAKIAGKIA-NH_2_
MtP-K8A-A7G-^15^N-A10	Ala10	KIAGKI**GA**I**A**GKIAKIAGKIA-NH_2_
MtP-a3-^15^N-A10	Ala10	KIAGK**AGA**I**A**GKIAKIAGKIA-NH_2_
MtP-a4-^15^N-A10	Ala10	KIAGK**AGA**I**A**GKIAKIA**L**KIA-NH_2_
MtP-A7G-G18L-^15^N-A10	Ala10	KIAGKI**G**KI**A**GKIAKIA**L**KIA-NH_2_
PGLa-^15^N-A10	Ala10	GMASKAGAI**A**GKIAKVALKAL-NH_2_

**Figure 8:**
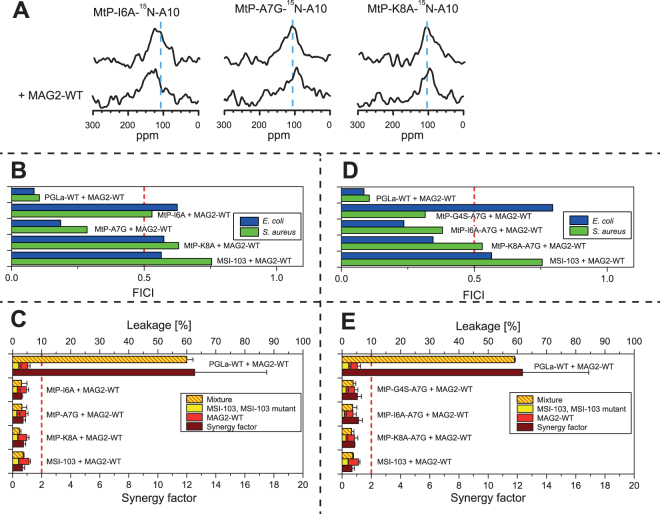
Synergy results for MSI-to-PGLa mutants. (**A**) ^15^N-NMR spectra of ^15^N-labeled MtP single mutants, alone or in 1:1 mixtures with MAG2-WT. None of the mutants show synergy with MAG2. (**B**) FICIs determined from combinations of MtP single mutants with MAG2-WT. A FICI below 0.5 (dotted line), indicating synergistic activity, is found only for MtP-A7G in both *E. coli* and *S. aureus*, but MtP-I6A and MtP-K8A did not show any synergy. (**C**) Leakage of MtP single mutants, alone or in 1:1 mixtures with MAG2-WT, showing no synergy for any of the MSI-103 mutants with MAG2. (**D**) FICIs determined from combinations of MtP double mutants with MAG2-WT. FICIs below 0.5 (dotted line), indicating synergistic activity, are found for all mutants in either *E. coli* or *S. aureus*, but only MtP-A7G showed synergy against both *E. coli* and *S. aureus*. (**E**) Leakage of MtP double mutants, alone or in 1:1 mixtures with MAG2-WT, showing no synergy for any of the MSI-103 mutants with MAG2.

Based on the PtM-a2 mutant, three single mutations were tested first: MtP-I6A, MtP-A7G and MtP-K8A. NMR showed no synergy for any of these peptides (Fig. 8A), and neither did leakage (Fig. 8C). Only the FICI (Fig. 8B) indicated that MtP-A7G was synergistic, but not MtP-I6A and MtP-K8A. The possible synergy against bacteria for MtP-A7G suggested that this residue was probably a step in the right direction, as had been expected, but this single mutation did not yet exhibit full synergy with all of our test methods. Therefore, double replacements were introduced in a second series of peptides, to make them more similar to PGLa.

All of the MtP double mutants included A7G, plus another mutation (I6A, K8A or G4S) within region a (Fig. 6A). ^15^N-NMR did not give useful results for these mutations, as no signal was detectable when these peptides were mixed with MAG2-WT. Several NMR samples were prepared fresh, and different experimental parameters were tried, but no signal was seen. The checker-board assay showed synergy for the MtP-A7G single mutant, hence it should be expected that also the double mutants should give synergy, but results were inconsistent (Fig. 8D). MtP-G4S-A7G showed synergy in *S. aureus* but not in *E. coli*, MtP-K8A-A7G showed synergy in *E. coli* but not in *S. aureus*, while MtP-I6A-A7G showed synergy in both cases. On the other hand, the sensitive leakage results were clear, as none of the double mutants showed any synergy (Fig. 8E).

**Figure 9:**
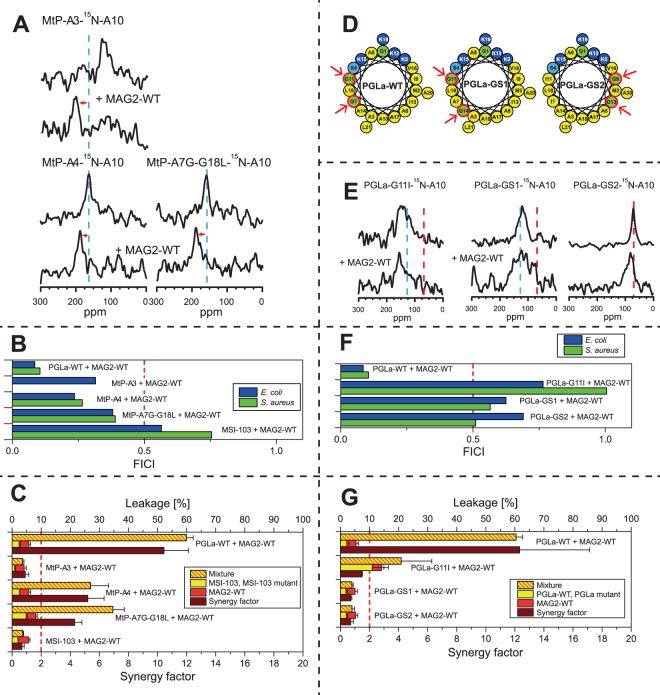
Synergy results for MSI-to-PGLa multiple mutants and PGLa glycine mutants. (**A**) ^15^N-NMR spectra of ^15^N-labeled MtP multiple mutants, alone or in 1:1 mixtures with MAG2-WT. All mutants show synergy with MAG2. (**B**) FICIs determined from combinations of MtP multiple mutants with MAG2-WT. FICIs below 0.5 (dotted line), indicating synergistic activity, is found for all mutants in both *E. coli* and *S. aureus*. (**C**) Leakage of MtP multiple mutants, alone or in 1:1 mixtures with MAG2-WT. MtP-a3 did not show synergy, but both MtP-a4 and MtP-A7G-G18L showed synergy with MAG2. **(D)** Helical wheels of glycine mutants of PGLa. In PGLa-WT, Gly7 and Gly11 appear to be part of a GxxxG motif. If one of these Gly is mutated, synergy is lost with MAG2. In PGLa-GS1, the GxxxG motif is changed to a GxxG motif and shifted along the helix. In PGLa-GS1, the GxxxG motif is retained but moved to the opposite face of the helix. The arrows indicate the corresponding Gly positions. (**E**) ^15^N-NMR spectra of ^15^N-labeled PGLa glycine mutants, alone or in 1:1 mixtures with MAG2-WT. All of the mutants have lost their synergy with MAG2. (**F**) FICIs determined from combinations of PGLa glycine mutants with MAG2-WT. PGLa glycine mutants do not show any synergy with MAG2-WT. (**G**) Leakage of PGLa glycine mutants, alone or in 1:1 mixtures with MAG2-WT. All of the mutants have lost the synergy with MAG2-WT. Even though PGLa-G11I showed a very high activity on its own, the synergy factor was low.

Since the goal was to create a fully synergistic peptide from MSI-103, according to all three methods, we synthesized a third series of peptides with multiple mutations. First, in MtP-a3 (Table 4), the three single mutations from the first generation of MtP peptides were combined. NMR (Fig. 9A) and the FICI (Fig. 9B) suggested that MtP-a3 was synergistic with MAG2, but leakage did not (Fig. 9C). Since leakage was the most sensitive method, it seemed that there was still a significant difference present between MtP-a3 and PGLa, which was critical for synergy in the leakage assay. In the reverse PtM approach above, leakage synergy was no longer seen for PtM-c1, while the other methods showed synergy. Thus, some residue in the c region might be involved in synergy, but with a weak interaction that gets noticed only in the leakage assay. Out of the four possibilities of mutations leading from MSI-103 to PGLa in region c, I16V, I20A and A20L did not seem to be likely candidates, because one hydrophobic residue was replaced by another. The most probable candidate was G18L, hence another mutant with the same three mutations as in MtP-a3 plus an additional G18L was prepared, called MtP-a4. This peptide showed perfect synergy with all three methods; NMR (Fig. 9A), FICI (Fig. 9B) and leakage (Fig. 9C), so a fully synergistic peptide was obtained by four mutations of MSI-103. However, from the previous results it seemed likely that the mutations I6A and K8A were not critical. Therefore, in a final design we created the double mutant MtP-A7G-G18L, which showed full synergy using all three methods. This important step proved that it was possible to convert MSI-103 into a synergistic partner for MAG2 by only two mutations within the MSI-103 sequence.

Remarkably, both of these mutations involved Gly residues, and we also note that the removal of Gly7 from PGLa had been the critical point towards losing its synergy. This observation points to Gly as being very important residues for the synergy, making it worthwhile to pursue additional investigations into the role of Gly, which is the subject of the next section.

### The role of glycine for synergy

So far, we have shown that the mutation G7A caused PGLa to lose its synergy with MAG2, while the opposite mutation A7G, together with one additional G18L mutation, made MSI-103 synergistic with MAG2. These findings point to an important role of Gly residues in the interactions that are responsible for synergy, one of which is located in the N-terminal a region and the other one in the C-terminal c region (see Fig. 6A). The latter has been found to have only a weak influence on synergy. In this region, charge interactions can occur, which are required to detect synergy in leakage experiments, but not in NMR or checker-board assays (Figs 3 and 4). Similarly, the G18L mutation is needed to observe synergy in leakage experiments, but not in the other two methods. Since the charge interaction seems most likely to involve E19 in MAG2 and K19 in PGLa, the role of L18 is probably to create an optimal environment for K19 to engage in an intermolecular salt bridge. When L18 is replaced with Gly, this could (i) destabilize the helix at the C-terminus, since Gly is known as a helix-breaker, and/or (ii) reduce the hydrophobicity of the segment and move it closer to the water phase, i.e. the helix would lose a hydrophobic anchor next to K19. It seems that under the conditions used in the NMR experiments, where peptides are forced to bind to the membrane and their concentration is high, the PGLa helix can still adopt the right position to interact with MAG2, even with mutations in its C-terminal region. Only under the conditions used for the vesicle leakage experiments, the hydrophobic side chain of L18 is required to observe synergy.

Gly7, on the other hand, seems to be intrinsically critical, as synergy was completely lost when it was mutated, even in the least sensitive NMR assay. As seen in the helical wheel projection of PGLa (Fig. 1), Gly7 and Gly11 are located on the same face of the helix. They are in fact part of a GxxxG motif, which has been shown to drive the dimerization of transmembrane helices^73–75^. If this GxxxG motif is involved in the complex formation of PGLa, then we would expect that also Gly11 should be important for synergy. This residue had not been singled out above, because it is also present in MSI-103. Now, we replaced it with Ile and found that indeed the PGLa-G11I mutant no longer showed any synergy with MAG2 in NMR (Fig. 9E), FICI (Fig. 9F), or leakage (Fig. 9G). Interestingly, PGLa-G11I had a very high leakage activity in the absence of MAG2, possibly because of its increased hydrophobicity, but it did not exhibit any synergy with MAG2.

To further investigate the GxxxG motif, two additional mutants were designed (Fig. 9D), as listed in Table 5. CD experiments showed that they all had the same secondary structure as PGLa-WT (Supplementary Fig. S6). Supplementary Tables S13–S16 list other data presented in the figures below. In these so-called GS (“glycine-shift”) mutants or isomers, pairs of amino acids in the PGLa-WT sequence were interchanged with one another, such that the total hydrophobicity of the helix was retained. In PGLa-GS1, the GxxxG motif is changed to a GxxG motif and shifted along the helix by the mutations G7A and A14G. Thus, there is now a GxxG motif with Gly11 and Gly14. In PGLa-GS2, the GxxxG motif is retained but moved to the opposite face of the helix by the mutations G7A, G11I, I9G, and I13G. Thus, there is now a GxxxG motif with Gly9 and Gly13. NMR (Fig. 9E), FICI (Fig. 9F), and leakage (Fig. 9G) all showed that PGLa-GS1 had lost its synergy with MAG2. The same was found for PGLa-GS2, and in this case the ^15^N-NMR data show some further interesting features.

**Table 5:** Peptides used to investigate the GxxxG motif in PGLa. Mutations derived from PGLa-WT are marked in bold, and ^15^N-labeled residues in bold underline.

Peptide	^15^N-label	Sequence
PGLa-WT	Ala10	GMASKAGAI**A**GKIAKVALKAL-NH_2_
PGLa-G11I-^15^N-A10	Ala10	GMASKAGAI**A** **I**KIAKVALKAL-NH_2_
PGLa-GS1-^15^N-A10	Ala10	GMASKA**A**AI**A**GKI**G**KVALKAL-NH_2_
PGLa-GS2-^15^N-A10	Ala10	GMASKA**I**A**G** **A** **I**K**G**AKVALKAL-NH_2_

According to all these results, PGLa obviously needs both Gly7 and Gly11 to show synergy with MAG2. Namely, when one of these Gly is mutated, ^15^N-NMR shows that the PGLa peak gets shifted away from 200 ppm by the presence of MAG2. The 200 ppm signal is characteristic of a peptide that is inserted in a transmembrane state. At a P/L of 1:50 in DMPC/DMPG membranes, as used here in the NMR experiments, PGLa-WT on its own shows a peak at approximately 130 ppm in the absence of MAG2. This signal corresponds to a tilted peptide, with an angle of around 125° between the helix axis and the membrane normal, as has been shown in detail also with ^2^H- and ^19^F-NMR^45–48^. Such a peak in the region of 120–150 ppm is also seen for all PGLa and MSI-103 mutants in this study when they do not engage in synergy – except for PGLa-GS2, which has a peak at 80 ppm. This characteristic signal is indicative of a peptide that lies flat on the membrane surface. Such tilt angle of 90° has been previously reported for PGLa and MSI-103 under different conditions, namely when they are at very low concentration in DMPC/DMPG, or when they are unable to insert into POPC/POPG bilayers^45,60^. Since all the other mutants of PGLa and MSI-103 (except for PGLa-GS2) possess a Gly either at position 7 or 11 (or both), it seems that one of these Gly’s is enough for the peptide to get into a T-state, while both are needed for synergy with MAG2. However, when both of these Gly’s are removed, as in PGLa-GS2, the peptide is found to lie flat on the membrane surface and cannot get into the T-state any more. It is of no help that there are now two Gly’s on the opposite face of the helix, in a similar position relative to the polar sector of the peptide (see helical wheel in Fig. 9D). This result supports our earlier proposal that an orientational change of PGLa or MSI-103 from the S-state into the T-state is indeed related to dimerization^46–48,59,76,77^. It also indicates that the native Gly residues are involved in the dimerization of these surface-bound amphiphilic peptides. Finally, our data also indicate that dimerization of PGLa is required for synergy with MAG2.

GxxxG was originally implied in the dimerization of hydrophobic transmembrane segments^73–75,78^. In amphipathic AMPs, the GxxxG motif has also been investigated. In the two-peptide bacteriocin lactococcin G from lactic acid bacteria, which has two peptide strands, there are GxxxG motifs present in both strands. In a mutation study, the Gly positions of these motifs were replaced with small (Ala or Ser) or large (Ile or Leu) residues and the MICs for the mutants in combination with the wild type of the other strand were investigated^79^. It was found that the Gly_7_xxxGly_11_ motif in the α strand was very sensitive even to small replacements, and such mutations reduced activity with about a factor of 10. On the other hand, the Gly_18_xxxGly_22_ motif was not sensitive even to large replacements. In the β strand, the Gly_18_xxxGly_22_ motif was only sensitive to large replacements but small replacements did not reduce activity^79^. Plasticins are Gly-rich AMPs from frogs, and in a recent study two of them, plasticin-B1 and plasticin-DA1, were investigated^80^. Both of them have several sequential GxxxG motifs, but no strong self-association was observed^80^. In that study, no attempt was made to mutate the Gly’s to see if the activity was affected^80^. Together with our results here, we can conclude that GxxxG motifs can be important for molecular contacts, but it is not enough for dimerization to have just any GxxxG motif, the exact position in the peptide is crucial. We can note that in PGLa, the G_7_xxxG_11_ motif is located in the region of the helical wheel in between the polar and hydrophobic faces (Fig. 9D). In plasticin-B1 and plasticin-DA1, the motifs are rather on the polar face^80^, which makes it less likely that contacts with another peptide can occur on the membrane surface in such an orientation that the GxxxG motifs directly face each other. But not even having the motif in the intermediate region is enough for synergy, since the G_9_xxxG_13_ motif in our PGLa-GS2 mutant did not result in a peptide which shows synergy with MAG2. We can conclude that the synergy depends in a very delicate way on exact peptide sequences.

It had been previously noticed from solid-state NMR results that lipids with a negative spontaneous curvature, like POPC/POPG, generally prohibit the tilting and insertion of amphiphilic peptides into the bilayer^45,60,81^. In such bilayers, PGLa on its own cannot reach the dimeric T-state at high concentration, but always remains as a monomer on the surface in the S-state. Likewise, PGLa in a 1:1 mixture with MAG2 does not flip into the I-state, but stays in the S-state. It thus seems that lipids with a negative spontaneous curvature prevent the tilting and dimerization of PGLa, and as a result also the synergy with MAG2 is hindered. Our glycine-shift studies also show that at least one of Gly7 and Gly11 must be present in the PGLa sequence to allow it to dimerize, even in favorable lipids like DMPC with a positive spontaneous curvature.

### Structure of the PGLa-MAG2 complex in membranes

In this study we have presented many new results that provide structural clues about the molecular interaction between PGLa and MAG2, when they form a synergistic complex in membranes. We will now summarize these results and combine them with previous studies of the two peptides, to see how it is possible to put together a plausible model of the PGLa-MAG2 architecture that is responsible for the synergy.

**Figure 10:**
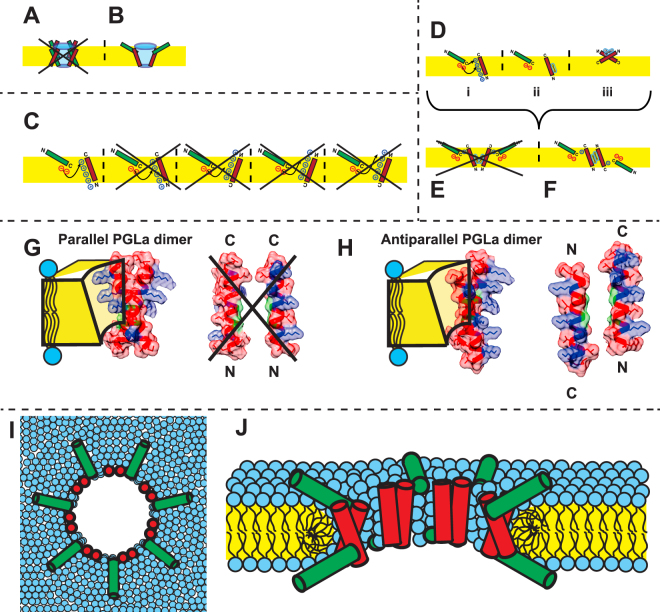
Models of heterodimers of PGLa (red) and MAG2 (green) in membranes. The orientation of the peptides in the membrane is shown according to solid-state NMR experiments. (**A**) The old proposed model, which could be excluded after solid-state NMR analysis of both components, which showed that PGLa is inserted in the membrane while MAG2 is not. (**B**) Model compatible with NMR and summarizes the current state of knowledge up to date. It is assumed that the heteromeric contact involves the C-termini of the two peptides, based on a cross-linking study and MD simulations. **(C)** Possible charge interactions. Most possibilities can be excluded from our mutation study, but the most likely point of contact is a weak electrostatic interaction between Lys19 of PGLa and Glu19 and/or the charged C-terminus of MAG2. The helixes are drawn in their correct orientations known from solid-state NMR. The N- and C-termini are labeled in such a way as to indicate clearly in which way a helix has been flipped or rotated to comply with the NMR constraints, which is especially important in the case of PGLa. **(D–F)** Model of a cross-section through a transmembrane pore formed by PGLa in synergy with MAG2. Panel (**D**) illustrates (**i**) the weak charge interactions between the C-termini of the two peptides, (**ii**) the transmembrane orientation of PGLa carrying the GxxxG motif on the appropriate face, and (**iii**) the proposed antiparallel dimer of PGLa in the absence of MAG2 (here, the GxxxG motif is actually hidden in the dimer interface). Combining this information, there are only two possibilities of constructing a heteromeric complex, either by parallel or antiparallel PGLa assembly. The model in (**E**) can be excluded, according to the arguments given below. Panel (**F**) shows the final architecture proposed here, consisting of an antiparallel membrane-spanning PGLa dimer that is in tail-to-tail contact with a surface-bound MAG2 monomer on each side of the membrane. (**G**) parallel and (**H**) antiparallel (right) dimers of PGLa in a membrane-spanning pore, with Gly residues (in green) facing each other. In the parallel arrangement, the positive charges on PGLa (in blue) are located on both faces of the dimer, making this unsuitable for lining a transmembrane pore. When arranging the two PGLa molecules as an antiparallel dimer, on the other hand, all charges are located on the same face of the dimer and could readily stabilize a water-filled pore. The space-filling models of the peptides as ideal α-helices were made using the Chimera software^100^. A hypothetical pore formed by tetrameric heterocomplexes is shown from the top (**I**) and as a cut through the membrane (**J**).

In earlier studies, the synergy between PGLa and MAG2 had been shown to be maximal for a 1:1 mixture of the peptides, suggesting that a heterodimer may be responsible for the synergy^25,30^. From a cross-linking study, parallel heterodimers seemed to be most likely, with the C-termini of both peptides in close contact^38^. An early ^2^H-NMR study had reported that PGLa, in the presence of an equimolar amount of MAG2 in a DMPC/DMPG bilayer at P/L = 1:50, assumed a transmembrane orientation with a tilt angle of 160° (i.e. the helix axis is tilted 20° from the membrane normal). In analogy to the symmetrical homodimeric complexes, it had then been suggested that also MAG2 could be inserted, leading to a pore model as shown in Fig. 10A ^49^. From ^15^N-NMR data, however, it was later found that MAG2 was not inserted in the presence of PGLa^45,50^, but a somewhat more tilted orientation (with a tilt of 120°, the helix axis is tilted 30° from the membrane plane) was seen in the presence of PGLa in a recent ^2^H-NMR study^44^, leading to a different possibility shown in Fig. 10B.

In the present study, we started off by hypothesizing that the negative charges close to the C-terminus of MAG2 may be able to interact electrostatically with some of the positive charges along the polar face of PGLa. Analysis of the corresponding series of charge mutants allowed us to exclude many possibilities, as illustrated in Fig. 10C. The most likely interaction is between Lys19 of PGLa and Glu19 and/or the charged C-terminus of MAG2. A recent MD simulation study also pointed to such interactions^64^. In that study, a re-orientation of PGLa was observed in the presence of MAG2, but when the negative charges were removed from MAG2, the re-orientation no longer occurred.

Our leakage data indicated a loss of synergy when the negative charges were removed from MAG2. However, checker-board assays showed that synergy was lost in *S. aureus* but not in *E. coli*, and NMR experiments showed that PGLa flipped into the inserted state even though both negative charges were removed from MAG2. It is possible that leakage, biological activity and peptide insertion work by different mechanisms, and that only leakage but not peptide insertion needs electrostatic interactions between the peptides. It also cannot be excluded that a different mechanism could be responsible for killing *S. aureus* versus *E. coli*. But since the vast majority of the data from the three different methods correlate so well (Fig. 2), despite the different time scales, local concentrations and lipid compositions involved (see Table 1), it is much more plausible to assume that the same molecular mechanism is responsible for all types of synergistic activity. Our interpretation is that PGLa and MAG2 form transmembrane pores that are stabilized by peptide-peptide interactions. Electrostatic interactions contribute to the total interactions and help to stabilize the pores. But these interactions cannot be very strong, because bacteria are killed also without them, and pore formation requires further interactions that are even more crucial.

The observation that MSI-103, having a sequence similar to PGLa, shows no synergy with MAG2, also points to electrostatic interactions not being critical, since MSI-103 contains all the positive charges found in PGLa, but this is not enough to display synergy with MAG2. The similarity of MSI-103 and PGLa initiated another mutation cycle. Successive rounds of mutations (Fig. S8A) led to the identification of Gly7 and Gly11 in PGLa as the key residues that are crucial for synergy. When one of these Gly’s was removed, synergy was lost according all three experimental methods. Another cycle of mutations was then performed to convert MSI-103 into a synergistic partner for MAG2, which was possible with only two mutations, A7G and G18L (Fig. S8B). The former introduces a GxxxG motif into MSI-103, yet this alone was not sufficient, because also Gly18 of MSI-103 had to be replaced by Leu to obtain synergy according to all methods. This means that the GxxxG motif is necessary but not enough for synergy, as also residues at the C-terminus have an influence on synergy.

Further mutations were performed to test the role of the GxxxG motif. It was found that synergy was lost when this motif was moved to the other side of the helix, and also when it was changed into a GxxG motif. It thus seems that both Gly residues are needed at their correct positions to support synergy between PGLa and MAG2. It is known that the GxxxG motif allows the formation of intermolecular Cα–H hydrogen bonds between adjacent helices that are in close contact, which stabilizes the dimer^82^. Such hydrogen bonds cannot be formed by an Ala mutant, which explains why this small change in side chain volume has such a pronounced effect on dimerization.

We can now summarize our findings in Fig. 10D, which shows the observed charge interactions between the C-termini of the two peptides, the known transmembrane orientation of PGLa, and the proposed antiparallel dimer of PGLa in the absence of MAG2. The electrostatic interactions are not very strong, but seem to contribute to the stability of the complex. It was previously reported that cross-linked peptides, carrying a disulfide bridge between the C-termini of PGLa and MAG2, induced more efficient vesicle leakage than a mixture of the peptides^38^, and therefore some contact can be expected here.

It has been previously shown that the two peptides show the highest synergy in a 1:1 molar ratio, which suggested that they form some kind of heterodimeric complex^25,30^. The newly identified GxxxG key motif in PGLa is present on the N-terminal half of the peptide, far from the C-terminus that was also shown to play a moderate role in synergy. When PGLa is aligned in its proper transmembrane orientation in the presence of MAG2, the GxxxG motif comes to lie far below the membrane surface, where it has no access to MAG2 which is suspended almost flat on the bilayer surface. It is therefore highly unlikely that the GxxxG motif makes any direct contact with MAG2. It is far more likely that this dimerization motif is needed to stabilize a transmembrane dimer of PGLa. GxxxG was previously shown to be involved in the dimerization of glycophorin A and other hydrophobic transmembrane helices^73–75,83^, hence it could play a similar role in the amphiphilic PGLa helix. However, we note that PGLa-G7A and PGLa-G11I, neither of which shows any synergy with MAG2, both induce leakage like PGLa-WT. In fact, PGLa-G11I on its own gives more leakage than PGLa-WT, but it does not show any synergistic enhancement of leakage in the presence of MAG2. This could indicate that PGLa on its own does not form transmembrane dimers, but rather works via an entirely different mechanism, e.g. like the transmembrane monomers described in a recent MD simulation^84^. That simulation did not investigate, however, whether such transmembrane monomer would support dye leakage, and thus be physically related to vesicle leakage experiments. Also, in such model our minor mutation like Gly → Ala would not be expected to have much effect. Therefore, the transmembrane monomer model is unlikely to represent the mechanism responsible for synergy.

Given that synergy is most efficient for an equimolar ratio, and PGLa very likely forms a transmembrane dimer, the corresponding PGLa/MAG2 complex should have a 2:2 stoichiometry. The lower part of Fig. 10E,F illustrates the two possible arrangements, by which the peptides could make contact via the GxxxG motif. Figure 10E illustrates the possibility of a parallel transmembrane dimer of PGLa, whose C-termini interact with two MAG2 molecules on the same side of the bilayer. In Fig. 10F, an antiparallel PGLa dimer is shown, in contact with one MAG2 molecule on each side of the membrane. If we look at the possible PGLa dimers in more detail (Fig. 10G,H), we see that the parallel dimer is less likely to participate in a water-filled transmembrane pore, since this dimer would carry positive charges on both opposite faces. On the other hand, in the antiparallel dimer all charges are located on the same face of the dimer, while the opposite face is hydrophobic. This arrangement is fully compatible with the formation of a toroidal pore, in which several dimers line the walls. This way, all charged residues will point towards the water-filled interior and possibly interact also with anionic lipid head groups to compensate for the total charge density. It has been shown that the optimal helix-helix crossing angle for the GxxxG motif is around 30–50°^82^. A solid-state ^2^H-NMR study showed that PGLa in the presence of MAG2 has a helix tilt angle of 22° between the helix axis and the membrane normal^16,49^. For an antiparallel dimer this orientation would give a helix-helix crossing angle of 44°, fitting perfectly with the theoretical results.

According to the antiparallel PGLa dimer model, MAG2 monomers should be present on both sides of the membrane. However, in the natural course of action when the antimicrobial peptides attack a bacterium, both PGLa and MAG2 will arrive from the same side and bind to the outer leaflet of the membrane. Such scenario is not a problem for our model, because it has been shown that MAG2 can form short-lived toroidal pores^25,85^. Instantaneously, some MAG2 molecules can translocate this way to the other side of the bilayer, to subsequently form a more stable heteromeric complex with PGLa that causes much greater damage to the cell. We thus propose that under synergistic action, PGLa and MAG2 assemble into a tetrameric complex as shown in Fig. 10F. Several such complexes will come together to form a long-lived membrane-spanning toroidal pore, which is responsible for the leakage of cellular contents and finally cell death. Both PGLa and MAG2 on their own are able to act as antibiotics and form pores, but when both of them are present at the same time, the pores are more stable and strong synergy is observed. A hypothetical cartoon model of such a pore, formed by tetrameric heteropeptide complexes, is shown in Fig. 10I,J. In a real fluid membrane, pores are probably dynamic and of different diameters, and obviously not as symmetric as the simple model pictured here. Furthermore, it is conceivable that instead of well-defined circular pores, the peptide complexes assemble in some other kind of long-range architecture, leading to peptide-lined bilayer rupture or to fragmentation into extended peptide-rimmed bicelles, Nonetheless, the molecular alignments, the peptide-peptide contacts and the peptide-lipid interactions deduced above, would be just the same as in the pore model presented here.

## Conclusions and Outlook

We present here a new model of the PGLa-MAG2 complex in a membrane, which can explain the synergistic antimicrobial activity of these two well-known peptides. From a large number of mutations, some key residues could be identified that are important for their functional interactions. In PGLa, most residues could be mutated without any loss of synergy, but Gly7 and Gly11 were found to be crucial for the synergy. Even when a single one of them was replaced with the next smallest amino acid, Ala, synergy was completely abolished. On the other hand, by introducing this GxxxG motif into the related antimicrobial peptide MSI-103, which by itself shows no synergy with MAG2, together with an additional G18L mutation it was possible to make MSI-103 synergistic with MAG2. This detailed understanding at a molecular level opens up the possibility to make also other cationic amphipathic helical peptides synergistic with MAG2 by similar mutations. A large number of such antimicrobial peptides are known.

So far, we have not yet found any residues in MAG2 that lead to a complete loss of synergy. A recent study suggested that replacing Phe residues with Ala gave a slightly reduced synergy, but only for some of the tested bacterial species^29^. In the present paper, the negative charges on MAG2 have been identified as important in the most sensitive vesicle leakage assay, but even when both charges were removed synergy was maintained in the biological assay and with solid-state NMR. A more specific interaction is quite likely present from the point of view of MAG2, but still needs to be identified.

## Materials and Methods

### Materials

Coupling reagents and Fmoc-protected amino acids were purchased from Iris Biotech GmbH (Marktredwitz, Germany) or Novabiochem (Merck Chemicals Ltd, Nottingham, UK). The ^15^N-labeled Ala was purchased from Cambridge Isotope Laboratories (Andover, USA) and was Fmoc-protected using Fmoc-Cl as previously described^86^. All solvents required for synthesis and HPLC purification were from Acros Organics (Geel, Belgium) or Biosolve (Valkenswaard, The Netherlands). Müller-Hinton medium and Resazurin used for the biological assays was purchased from Becton, Dickinson and Company (Sparks, USA) and from Sigma-Aldrich (Taufkirchen, Germany). The microtiter plates were from Thermo Scientific (Waltham, USA). UV-grade chloroform and methanol used for NMR sample preparation were obtained from VWR International (Bruchsal, Germany). The lipids 1,2-dimyristoyl-*sn*-glycero-3-phosphatidylcholine (DMPC), 1,2-dimyristoyl-*sn*-glycero-3-phosphatidylglycerol (DMPG), 1-palmitoyl-2-oleoyl-*sn*-glycero-3-phosphatidylcholine (POPC), 1-palmitoyl-2-oleoyl-*sn*-glycero-3-phosphatidylglycerol (POPG), 1′,3′-bis[1,2-dioleoyl-*sn*-glycero-3-phospho]-*sn*-glycerol (TOCL), and 1,2-dioleoyl-*sn*-glycero-3-phosphoethanolamine-*N*-(lissamine rhodamine B sulfonyl) (Rhod-PE) were obtained from Avanti Polar Lipids (Alabaster, USA) or NOF Europe (Grobbendonk, Belgium). ANTS and DPX were purchased from Invitrogen (Eugene, USA). Triton-X-100, HEPES, and Sephacryl 100-HR were from Sigma-Aldrich Chemie GmbH (Steinheim, Germany).

### Peptide synthesis

MSI-103 with the sequence (KIAGKIA)_3_-NH_2_, PGLa with the sequence GMASKAGAIAGKIAKVALKAL-NH_2_, and all analogs derived from these peptides were synthesized with ^15^N-Ala at position 10, at a 100 µmolar scale on an automated Syro II peptide synthesizer (MultiSynTech, Witten, Germany). The ^15^N-labeled amino acid was coupled manually in DMF by using 2.0 equivalents excess of Fmoc-^15^N-amino acids in the presence of 1-hydroxybenzotriazole (HOBt), 1-benzo-triazole-1-yl-*N*,*N*,*N*′,*N*′-tetramethyluronium hexafluorophospate (HBTU), and *N*,*N*-diisopropylethylamine (DIPEA) as a base, in a molar ratio of 2:2:2:4, respectively. Magainin 2 (MAG2) with the sequence GIGKFLHSAKKFGKAFVGEIMNS-OH, and all analogs thereof were synthesized on an automated Liberty 1 microwave peptide synthesizer (CEM, Kamp-Lintfort, Germany) at a 250 µmolar scale. We found that the synthesis of MAG2 was generally more difficult in terms of coupling efficiency and yielded less product (~10% yield) than for PGLa or MSI-103 (~20-50%). The crude peptides were purified via HPLC using C18 reverse phase HPLC columns, with water/acetonitrile gradients, each containing 5 mM HCl as an ion pair agent. The purity of the peptides was confirmed using an LC-MS system equipped with an Agilent 1100 Series LC-system coupled to an ESI µ-TOF mass spectrometer from Bruker Daltonics.

### Biological assays

#### Minimum inhibitory concentration (MIC)

The antimicrobial activity of the peptides was investigated with a standard MIC assay^87,88^ on one Gram positive (*Staphylococcus aureus*, DSM 1104, ATCC 25923) and one Gram negative bacterial strain (*Escherichia coli*, DSM 1103, ATCC 25922). Bacteria were grown in Müller-Hinton medium (MH) at 37 °C and 220 rpm. Overnight cultures were inoculated from a single colony and grown for approximately 16 h. Day cultures were grown for approximately 3 h until they reached the exponential growth phase. These suspensions were diluted with MH-medium to a concentration of 10^6^ colony-forming units (CFU) per ml. The microtiter plates (96 wells of 100 µL) were filled with 50 µL of MH, and serial 2-fold dilutions of each peptide were arranged in triplets along the columns. Since the peptide stocks did not contain MH, the first row with MH was prepared with double concentration to reach the same medium concentration in each well. Two columns were filled with medium only, to serve as a positive control (bacteria), and as a negative control (no bacteria) to check for contamination of the medium. The bacterial suspension was added in 50 µL aliquots to each well, except for the negative control, to reach a final concentration of 5 × 10^5^ CFU/ml. The plates were incubated for 22 h at 37 °C. To each well 20 µL of 0.2 mg/ml Resazurin solution was added and incubated for another two hours. The MIC value was determined visually, as the lowest peptide concentration which inhibited bacterial growth in at least two out of three columns.

#### Checker-board assay

To visualize synergy between different peptides, a two-dimensional dilution assay was used^89,90^. The same bacterial strains were used as in the MIC assay. The procedure of preparing the bacterial suspensions was analogous to the MIC assay, but with a higher final concentration because the volumes in the two setups are different. The main plate (MP) was filled with 40 µl of MH, and serial 2-fold dilutions of the peptide were arranged in each row (8 rows × 7 columns). As described above, the first column was filled with doubly concentrated medium. On another plate (HP, help plate) a second peptide dilution assay was performed, but orthogonal to the main plate. The last row was left empty (7 rows × 8 columns). 40 µL of each well of the HP were transferred to each well of the MP (A1 → A1, etc.). Two columns were filled with medium only, to serve as a positive control (bacteria), and as a negative control (no bacteria) to check for contamination of the medium. The bacterial suspension was added in 20 µL aliquots to each well, except for the negative control, to reach a final concentration of 5 × 10^5^ CFU/ml. The plates were incubated for 22 h at 37 °C. To each well 20 µL of 0.1 mg/ml Resazurin solution was added and incubated for another two hours. From these plates, it is possible to determine the MIC value for each of the two peptides, as well as the inhibitory values of the mixtures with 7 × 7 different peptide ratios. From these values, fractional inhibitory concentration indices (FICIs) were calculated with the following formula^89,91^:

1 $${\rm{FICI}}=[{\rm{MIC}}{({\rm{A}})}^{{\rm{comb}}}/{\rm{MIC}}{({\rm{A}})}^{{\rm{alone}}}]+[{\rm{MIC}}{({\rm{B}})}^{{\rm{comb}}}/{\rm{MIC}}{({\rm{B}})}^{{\rm{alone}}}]$$

In this formula, MIC(A)^alone^ is the MIC value determined for peptide A alone, and MIC(A)^comb^ is the concentration of peptide A at the MIC determined for the combination. The same holds for peptide B. FICI values below 0.5 are taken to indicate a clear synergistic effect, values between 0.5 and 2 an additive effect, and values above 2 an antagonistic effect^89,92^.

### Circular dichroism spectroscopy

All CD measurements were performed on a J-815 CD spectropolarimeter (Jasco, Tokyo, Japan). The average of three measurements recorded from 260 nm to 180 nm gave the final CD spectrum. The measurements were performed in continuous scanning mode with a scanning speed of 10 nm/min, 1 nm spectral bandwidth, 0.1 nm data pitch, and 8 s response time of the detector. Because the MSI-103, PGLa and MAG2 sequences do not contain any aromatic Trp or Tyr residues, an exact determination of the peptide concentration via UV absorption spectroscopy was not possible. To calculate the mean residue ellipticity values (MRE), the peptide concentration was calculated from the weight and the added buffer volume. Differences in spectral intensities can be caused by different salt concentrations, impurities, weighing and pipetting errors. MRE values were calculated with the following formula^93^:

2 $${{\rm{\theta }}}_{{\rm{MRE}}}={\rm{MRW}}\times {\rm{\theta }}/(10\times {{\rm{c}}}_{{\rm{g}}}\times {\rm{d}})$$

where θ is the ellipticity (in mdeg), MRW is the mean residue (amino acid) weight in g mol^−1^, c_g_ is the concentration in mg ml^−1^, and d is the optical path length (in cm). θ_MRE_ has the unit deg cm^2^ dmol^−1^. The temperature was 25 °C in phosphate buffer, and 30 °C in the presence of DMPC/DMPG (3:1) vesicles at a P/L of 1:50. Vesicles were prepared as previously described^24^. To obtain a P/L of 1:50 in the vesicle samples, we used a lipid concentration of 1.5 mM and a peptide concentration of 0.03 mM. In buffer solution, the peptide concentration was 0.03 mM.

### Solid state NMR

#### NMR sample preparation

Lipids and peptides were co-dissolved in suitable amounts of organic solvents, spread onto glass plates, and after removal of the solvents hydrated at 48 °C at 96% relative humidity. Usually 0.5–0.6 mg of the ^15^N-labeled peptide was used, equimolar amounts of the partner peptide, and appropriate amounts of lipids to obtain the desired P/L ratio of 1:50. As a lipid system we used DMPC/DMPG (3:1) throughout. ^15^N-labeled MAG2 or PGLa was solubilized in MeOH (40 µL) and water (20 µL). In samples with two peptides, both peptides were dissolved in water (10 µL), and MeOH was added (40 µL) to the solution with the unlabeled peptide. Lipids were dissolved in 120 µL chloroform and 160 µL methanol, and added to the peptide solution. The lipid vessel was rinsed with additional chloroform (50 µL) which was also added to the peptide solution. The final container was always the one containing the labeled peptide. After 5 min sonification, the resulting clear solution was uniformly spread onto typically 23 glass plates of size 7.5 mm × 9 mm × 0.08 mm (Marienfeld Laboratory Glassware, Lauda-Königshofen, Germany). After evaporation of the organic solvent under reduced pressure, the glass plates were stacked and hydrated for 16–24 h at 48 °C in a humid chamber, obtained from a saturated solution of K_2_SO_4_, giving 96% relative humidity^94^. To keep the sample fully hydrated throughout the measurements, it was wrapped in parafilm and polyethylene foil. The sample quality was checked by monitoring the degree of lipid orientation by using solid state ^31^P-NMR.

#### NMR experimental details

^1^H-^15^N cross polarization experiments using a CP-MOIST pulse sequence^95^ were performed on an Avance III Bruker NMR spectrometer (Bruker Biospin, Karlsruhe, Germany) with a wide-bore 500 MHz or 600 MHz magnet. The spectra were acquired using a double-tuned probe with a low-E flat-coil resonator (3 mm × 9 mm cross section), employing typically a ^1^H and ^15^N radiofrequency field strength of 65 kHz during cross polarization, and 36 kHz ^1^H SPINAL16 decoupling^96^ during acquisition. 10000 to 30000 scans were accumulated with mixing times of 500 or 1000 µs. The acquisition time was 10 ms and the recycle time 3 s. The ^15^N chemical shift was referenced using a dry ammonium sulfate powder sample, where the ^15^N chemical shift was set to 26.8 ppm. To check the sample quality and degree of orientation, ^31^P-NMR measurements were performed using a Hahn echo sequence^97^ with a typical 90° pulse of 3.9 µs and a 35 µs echo time, with ^1^H SPINAL64 decoupling during acquisition. Typically 256 scans were accumulated. The acquisition time was 10 ms and the recycle time 1 s. All NMR samples were measured at 35 °C. The temperature of the sample inside the NMR probe was calibrated using a methanol sample^98^.

### Vesicle leakage assay

Leakage of small unilamellar vesicles induced by the peptides was studied using a standard fluorescence assay^99^.

#### Vesicle preparation

The appropriate lipids for each experiment were dissolved in CHCl_3_, together with 0.02 mol-% Rhodamine-PE (Rh-PE). The solvent was removed, and the dried lipids were hydrated with 10 mM HEPES buffer (pH = 7.5) containing 12.5 mM ANTS, 45 mM DPX, and 50 mM NaCl. This stock suspension was subjected to 10 freeze-thaw cycles and then frozen. Appropriate aliquots of the stock lipid suspension were unfrozen each day and extruded (41 times) through polycarbonate membranes with 100 nm pore size, and stored at room temperature overnight. The fluorophore and quencher outside the vesicles were removed by size exclusion chromatography using spin-columns (Pierce centrifuge column, 2 ml, Life Technologies GmbH, Darmstadt, Germany) filled with Sephacryl 100-HR (2 min, 1500 × g). For elution, a 10 mM HEPES buffer (pH 7.5) was used, containing 155 mM NaCl to balance the osmolarity. After extrusion and removal of the external dye and quencher, the lipid concentration usually decreases. Therefore, before each set of measurements a rhodamine spectrum was recorded to determine the actual concentration of lipids, which was referenced to a spectrum taken of vesicles made by sonification without any subsequent treatment.

#### Fluorescence measurements

Fluorescence experiments were performed on a FluoroMax2 spectrofluorimeter (HORIBA Jobin Yvon, Unterhaching, Germany) equipped with a thermostated sample holder and magnetic stirrer, using an excitation wavelength of 355 nm, an emission wavelength of 508 nm, and slit widths of 5 nm. The peptide solutions were placed in elution buffer in a 1 cm standard cuvette at 30 °C. 100 s after the start of the measurement lipid vesicles were added, and the intensity of the fluorescence at 508 nm was recorded. The leakage was measured during 600 s after addition of vesicles, and the measurement was terminated after addition of Triton-X-100 (0.25% v/v), which dissolved the vesicles, and yielded 100% leakage. To determine the background signal of the vesicles themselves, several blanks were recorded per measurement set. From the leakage values of the peptides alone and of the mixture of two peptides a synergy factor SF was calculated:

3 $${\rm{SF}}={\rm{L}}({\rm{A}}+{\rm{B}})/[{\rm{L}}({\rm{A}})+{\rm{L}}({\rm{B}})]$$

Here, L(A) is the leakage found with peptide A alone, L(B) is the leakage found with peptide B alone, and L(A + B) is the leakage found with the mixture of peptides A and B. In all cases the P/L was the same for both peptides, alone and in the mixture, so that in the mixture the total P/L was twice that of the individual peptide measurements. Since the leakage was very sensitive to small changes in peptide and lipid concentration, the nominal P/L was slightly adjusted each day to get the same degree of leakage for the 1:1 mixture of wild type peptides, which was the internal standard used to guarantee comparable conditions on each measurement day. Measurements were repeated three times and average values and standard deviations calculated for the leakage and the synergy factors. An average SF ≥ 2 was taken to indicate synergy.

### Data Availability Statement

All relevant data are within the article and its Supplementary Information file.

## Electronic supplementary material

Supplementary Information
