# Supplementary material for: Molecular mechanism of synergy between the antimicrobial peptides PGLa and magainin 2
Source: Sci Rep. 2017 Oct 13;7:13153. doi: 10.1038/s41598-017-12599-7 (PMC5640672; doi:10.1038/s41598-017-12599-7)
Supplement: Supplementary file 1 — Supplementary Information [file 41598_2017_12599_MOESM1_ESM.doc]

SUPPLEMENTARY INFORMATION

Molecular mechanism of synergy between the antimicrobial peptides

PGLa and magainin 2

Jonathan Zerweck,^1^ Erik Strandberg,^2^ Olga Kukharenko,^1^ Johannes Reichert,^2^ Jochen Bürck,^2^ Parvesh Wadhwani,^2^ and Anne S. Ulrich^1,2^,*

^1^ Karlsruhe Institute of Technology (KIT), Institute of Organic Chemistry, Fritz-Haber-Weg 6, 76131 Karlsruhe, Germany; ^2^ KIT, Institute of Biological Interfaces (IBG-2), POB 3640, 76021 Karlsruhe, Germany.

*Correspondance: anne.ulrich@kit.edu

**Correlation between the three used methods**

The three methods measure different aspects of the peptides in the membranes. FICI is a way of determining synergy for inhibiting bacterial growth, leakage shows synergy in inducing leakage of vesicles, and ^15^N-NMR shows the orientation of ^15^N-labeled PGLa on its own or in the presence of MAG2. We assume that bacterial killing and leakage is due to formation of pores, and that the inserted state of PGLa is also indicating the formation of a stable pore. All three methods are therefore assumed to show the same thing: formation of pores. Therefore, a mutation which induces or blocks the formation of pores could be identified with any of the methods, and all methods should show the same result. If this assumption is wrong, we should see contradicting results. However, as seen in **Fig. 2** in the main text, there is a strong correlation between the results.

As a first check, we can look at the FICI values for *E. coli* and *S. aureus*. 29 different peptide pairs were tested, and in 24 cases, the same result (synergy or no synergy) is found for both strains. In 4 cases, synergy was found in *E. coli* but not in *S. aureus*, and in only one case synergy was found in *S. aureus* but not in *E. coli*.

As a second test, we compare leakage in POPC/POPG and POPE/POPG/TOCL (For simplicity we will abbreviate the systems to PC/PG and PE/PG). 24 peptide pairs were tested in both lipid systems, the same result is found in 23 cases, and only in one case was synergy found in PE/PG but not in PC/PG. (And in this case the calculated synergy factor in PE/PG is 2.5 ± 0.5, very close to our cutoff of 2.0) Since we in general found a larger synergy factor in PE/PG of around 18, compared to around 11 in PC/PG, it is reasonable that in some border line case synergy could be found in PE/PG but not in PC/PG. So these results are almost identical.

If we now compare the main methods, 23 peptide pairs were studied with FICI and NMR. In 19 cases, the results were the same. In 3 cases, the results were partially the same (i.e. the FICI showed different results for the two strains: in two cases FICI in *E. coli* gave the same result as NMR, in one case *S. aureus*). Only in one case FICI showed synergy but NMR did not.

29 peptide pairs were studied with FICI and leakage, and in 18 cases the result was the same, in 5 cases partially the same (in one case FICI in *E. coli* gave the same result as leakage, in four cases for *S. aureus*). In 6 cases FICI showed synergy but leakage did not.

NMR and leakage were used on 25 peptide pairs, in 18 cases the same result was obtained, in 6 cases NMR showed synergy but leakage did not, and in one case NMR showed synergy whereas leakage showed synergy in PE/PG but not in PC/PG.

One conclusion is that leakage is the method which is most sensitive to mutations of the peptides. Compared with NMR, in no case was synergy found in leakage, but not in NMR. Likewise, there is no case where synergy was found in leakage but not in FICI for at least one bacterial strain. On the other hands, there are several cases where there is no synergy for leakage, but leakage is found using FICI or NMR.

Another, somewhat unexpected, conclusion is that synergy as determined from inhibition of bacterial growth shows a stronger correlation with NMR results than with leakage results, even though the NMR sample conditions are seemingly far removed from those of living bacteria. Or in other words, for a given peptide pair, an inserted state found in NMR in DMPC/DMPG is a better predictor of synergy against bacteria, than a synergistic effect in leakage in vesicles of a lipid mixture mimicking bacterial membranes.

In summary, and as shown in **Figure 2** in the main text and **Figure S7** below, there is a strong correlation between the results of the three methods, strongly suggesting that indeed they all measure the same underlying pore-forming ability of the peptide pairs. But there is a difference in the sensitivity of the methods to peptide mutations. This could be due to a difference in peptide-peptide interaction strength between the methods, and to assess this the differences between the methods are now discussed in more detail.

**Comparison of the three used methods**

We here want to discuss the particularities of the three different methods used in this study to determine synergy. NMR experiments give information about the orientation of the peptides in a membrane, and synergy is assumed to be related to the insertion of PGLa in the presence of MAG2, presumably forming a membrane-spanning pore complex. These experiments are performed in DMPC/DMPG (3:1) lipid bilayers, at a relatively high peptide concentration of P/L=1:50 for each peptide. This lipid system and P/L ratio were chosen because insertion of PGLa had been previously observed under these conditions^1-3^. The macroscopically oriented membrane samples used here have a limited degree of hydration, where no excess of bulk water is present, such that all peptides are forced to bind to the membrane^4^.

Vesicle leakage experiments, on the other hand, are performed in POPC/POPG (3:1) vesicles at a lower P/L of around 1:160 for each peptide, with a large excess of water. The lower overall P/L ratio, plus the fact that some peptides may remain unbound in solution, mean that the effective peptide concentration in the membrane is lower in these experiments than in the ^15^N-NMR samples. Furthermore, POPC/POPG has a negative spontaneous lipid curvature, which has been shown to disfavor the inserted state of PGLa, while DMPC/DMPG has a positive spontaneous curvature that favors insertion^1, 5, 6^.

Comparing leakage in POPC/POPG (1:1) and in POPE/POPG/TOCL (72:23:5), the latter system has an even stronger negative spontaneous curvature (due to PE and CL lipids with a small head group), which disfavors insertion of peptides. These properties explain why a higher peptide concentration (P/L ≈ 1:30) was needed in POPE/POPG/TOCL than in POPC/POPG (P/L ≈ 1:160) to obtain the same extent of leakage.

Thus, the NMR experiments are performed under conditions favoring pore formation, whereas leakage experiments are done under conditions where pore formation is less favorable. This is actually necessary for the experiments.

NMR picks up the orientation of all PGLa peptides in the sample at the same time, and it only gives a signal for inserted peptides when these are predominantly arranged a stable inserted state. This must therefore be done under conditions favoring a stable pore.

On the other hand, leakage will occur even if only a small proportion of peptides are engaged in pore formation, and such pores may only be transient. To observe the synergy, we need to do experiments under conditions were each peptide by itself gives only a small amount of leakage (a few percent), because when peptides are mixed the leakage is much higher (by a factor 10-20), and to get a reliable synergy factor the leakage under these conditions must still be well below 100%.

Thus, leakage experiments are sensitive to the ability of a peptide to form a few transient pores, whereas NMR is a bulk measure and will only be sensitive to the ability of peptides to form stable, long-lived pores. Under identical conditions, we can expect different results for the two methods.

It may seem best if NMR and leakage experiments are performed under identical conditions so results can be directly compared. But when NMR experiments were performed in POPC/POPG or POPE/POPG/TOCL, no stable insertion was found for PGLa in the presence of MAG2^7^, since these conditions are unfavorable for insertion. On the other hand, when leakage experiments were done with DMPC/DMPG vesicles, considerable baseline leakage was found even without any peptides (data not shown), meaning that those vesicles are inherently too leaky to be used in this assay.

For a balanced comparison, we can thus expect that leakage experiments, which are performed under conditions disfavoring pore formation, will be very sensitive to peptide-peptide interactions, and a small reduction in the peptide-peptide interaction strength could lead to a destroyed synergy. On the other hand, NMR experiments are performed under conditions favoring pore formation, and therefore a pore may form even if the peptide-peptide interactions are reduced, so that major disruptions of interactions are needed to block the pore formation. Therefore, it is not surprising that some of the mutations studied resulted in a loss of synergy in leakage but not in NMR. The opposite case was never observed.

The checker-board assay, finally, which is used to determine the FICIs, is a biological experiment that can be influenced by slight changes in the environment, affecting bacterial growth, and is therefore less reproducible. Also, since two-fold dilution series are used for both peptides, only approximate values can be determined, and a change in FICIs with a factor two or even four might not even be significant. Thus, we should be aware that the error in FICIs is larger than in the other methods, but it is an important method, since it is used to study synergy in living bacteria, and the main interest in the peptides is due to their ability to kill bacteria. From the comparison of the results, we observe that synergy as measured by FICI is less sensitive to mutations than synergy in leakage. The conditions of the checker-board experiment, with a very high peptide concentration (it has been estimated that there is 100 or more peptides per lipid at the MIC^8^) which should favour pore formation, but a large amount of lipids like PE and CL with a negative spontaneous curvature which should disfavour pores (but it can be noted that there are parts of bacterial membranes with a positive curvature, like the bacterial septum) seems to make the synergy measured with this method having a sensitivity to mutations more similar to that of NMR than of leakage, and in those cases where there is a different result between FICI and leakage, always less synergy is found in the leakage experiment.

For the purpose of our study, we can now combine the strengths of the different methods. When we try to find amino acids responsible for synergy, we are doing two kinds of experiments. When we try to remove synergy, we want to find residues that completely abolish it, so that we want all three methods to show no synergy, also the more robust synergy seen in NMR. On the other hand, when we want to find a new synergistic peptide, we want all three methods to show synergy, even the most sensitive synergy seen in leakage.

**References**

1. Strandberg, E., et al. Influence of hydrophobic residues on the activity of the antimicrobial peptide magainin 2 and its synergy with PGLa. *J. Pept. Sci.* **21**, 436-445 (2015).

2. Strandberg, E., Tremouilhac, P., Wadhwani, P. & Ulrich, A.S. Synergistic transmembrane insertion of the heterodimeric PGLa/magainin 2 complex studied by solid-state NMR. *Biochim. Biophys. Acta* **1788**, 1667–1679 (2009).

3. Tremouilhac, P., Strandberg, E., Wadhwani, P. & Ulrich, A.S. Synergistic transmembrane alignment of the antimicrobial heterodimer PGLa/magainin. *J. Biol. Chem.* **281**, 32089-32094 (2006).

4. Tremouilhac, P., Strandberg, E., Wadhwani, P. & Ulrich, A.S. Conditions affecting the re-alignment of the antimicrobial peptide PGLa in membranes as monitored by solid state ^2^H-NMR. *Biochim. Biophys. Acta* **1758**, 1330-1342 (2006).

5. Strandberg, E., Tiltak, D., Ehni, S., Wadhwani, P. & Ulrich, A.S. Lipid shape is a key factor for membrane interactions of amphipathic helical peptides. *Biochim. Biophys. Acta* **1818**, 1764-1776 (2012).

6. Salnikov, E.S. & Bechinger, B. Lipid-controlled peptide topology and interactions in bilayers: structural insights into the synergistic enhancement of the antimicrobial activities of PGLa and magainin 2. *Biophys. J.* **100**, 1473-1480 (2011).

7. Strandberg, E., Zerweck, J., Wadhwani, P. & Ulrich, A.S. Synergistic insertion of antimicrobial magainin-family peptides in membranes depends on the lipid spontaneous curvature. *Biophys. J.* **104**, L9-11 (2013).

8. Wimley, W.C. & Hristova, K. Antimicrobial peptides: successes, challenges and unanswered questions. *J. Membr. Biol.* **239**, 27-34 (2011).

SUPPLEMENTARY FIGURES

| 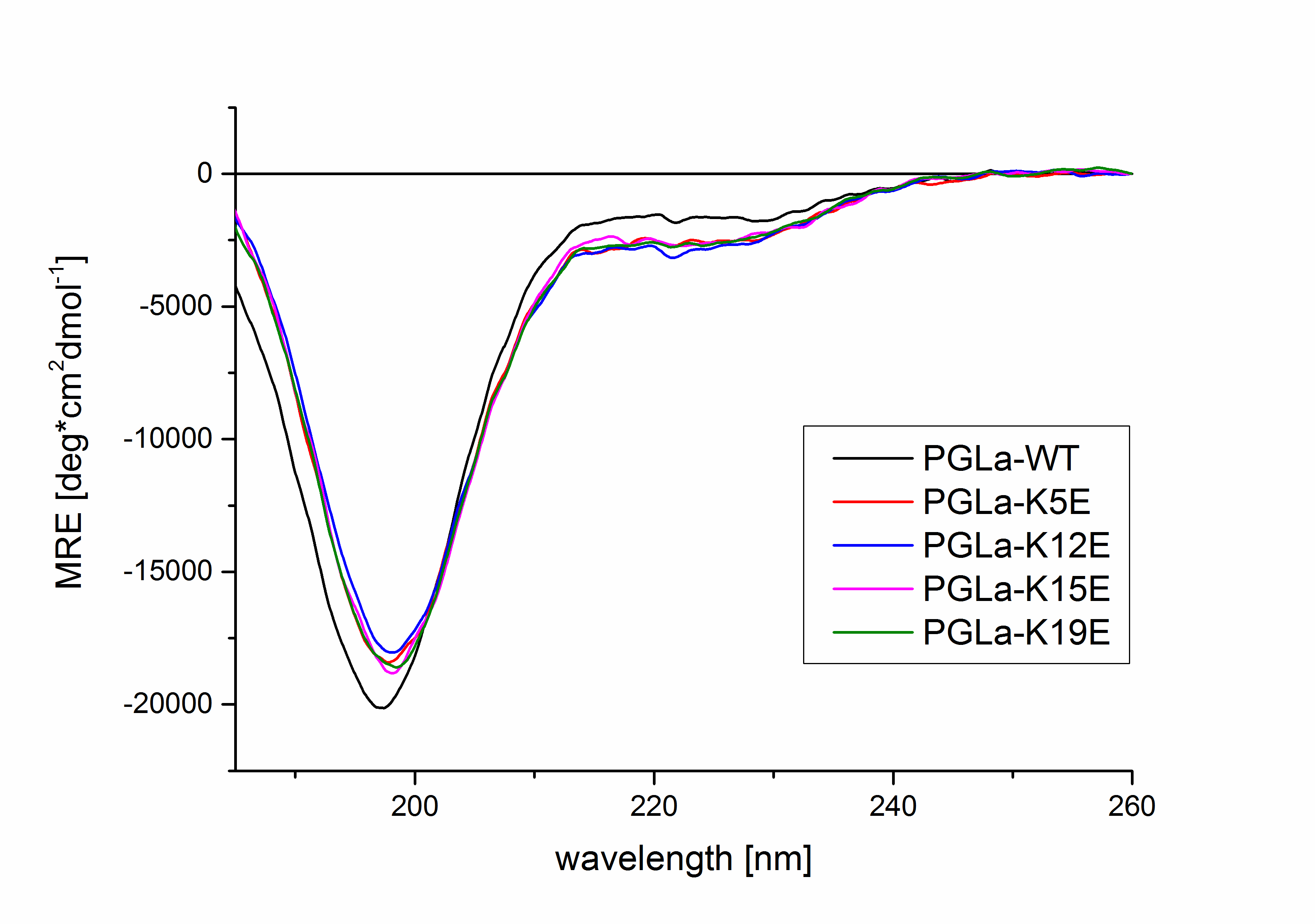 | 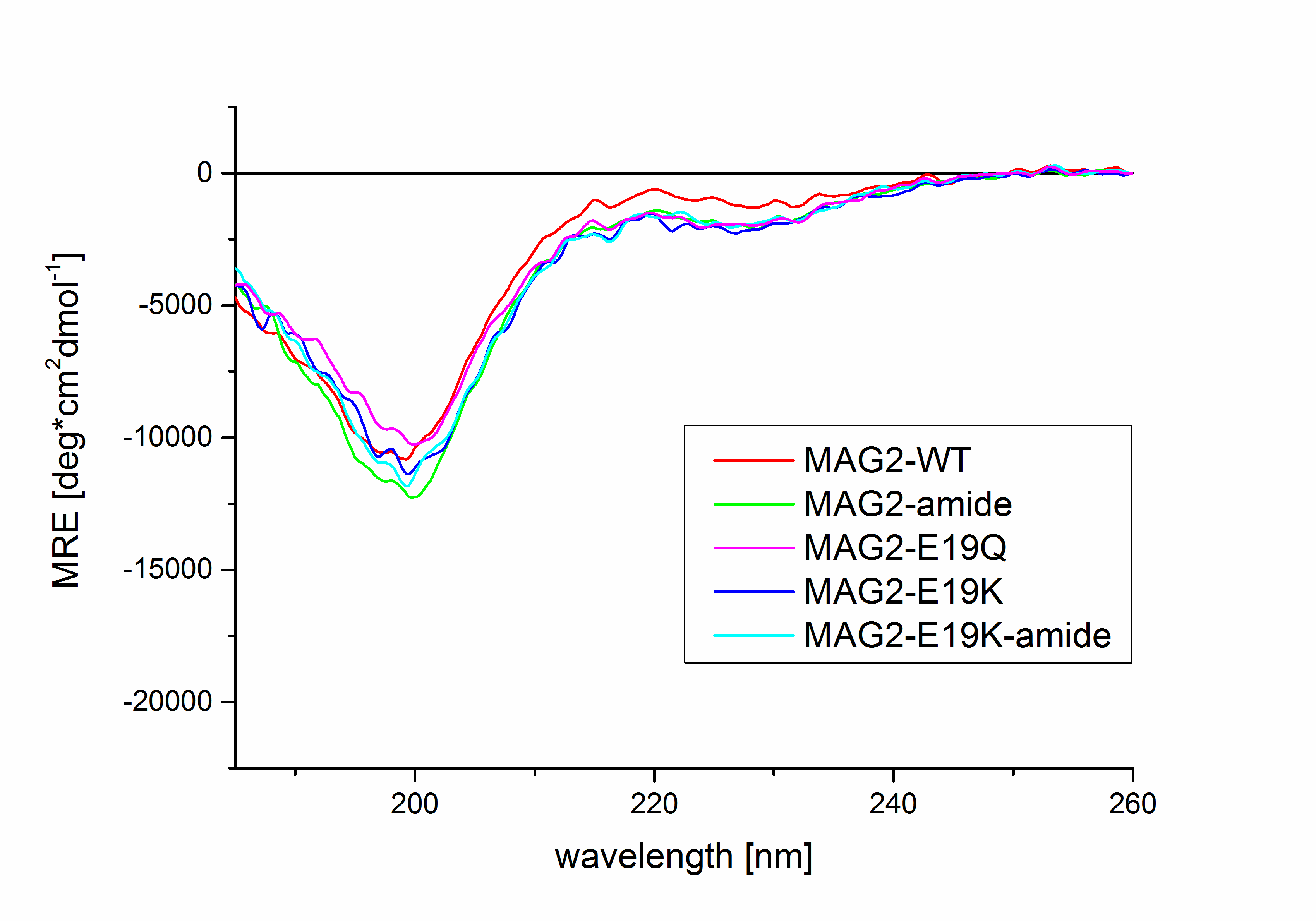 |
| --- | --- |

**Figure S1. CD spectra of charge mutants in phosphate buffer.** All peptides show spectra typical of random coils with a minimum around 198 nm.

| 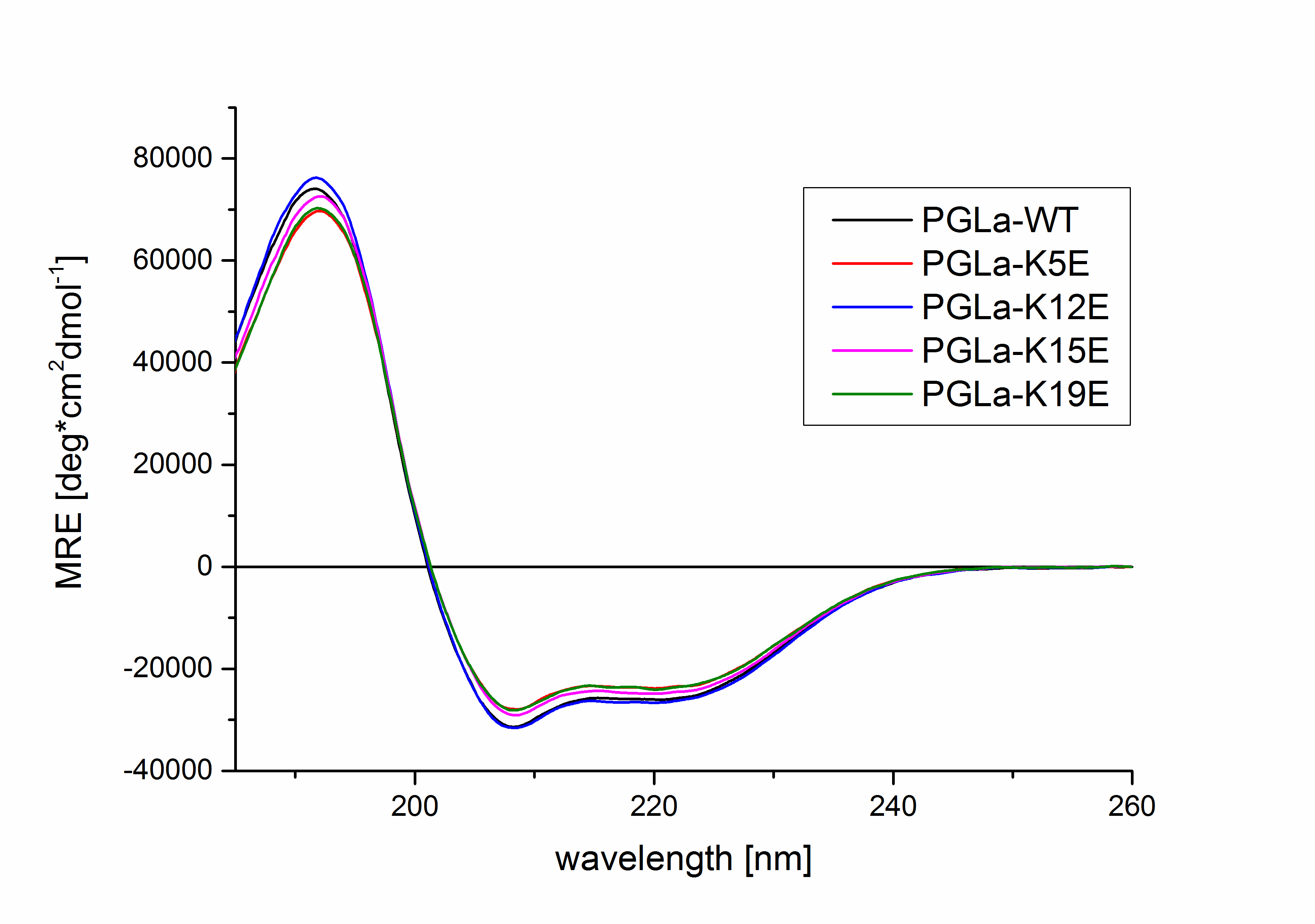 | 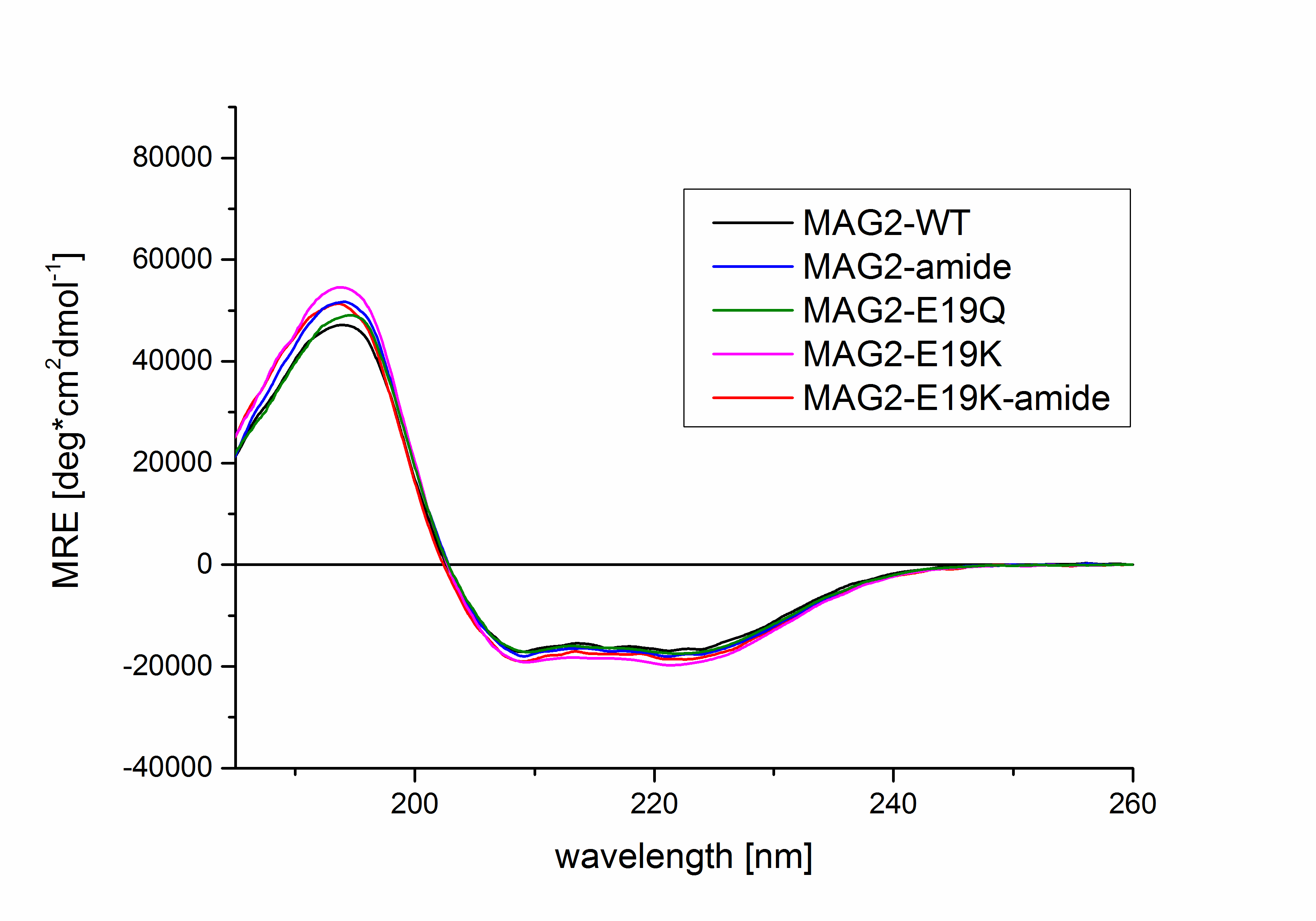 |
| --- | --- |

**Figure S2. CD spectra of charge mutants in DMPC/DMPG vesicles.** All peptides show spectra typical of α-helical conformation, with a positive band around 192 nm and two negative bands at 208 and 222 nm.

| 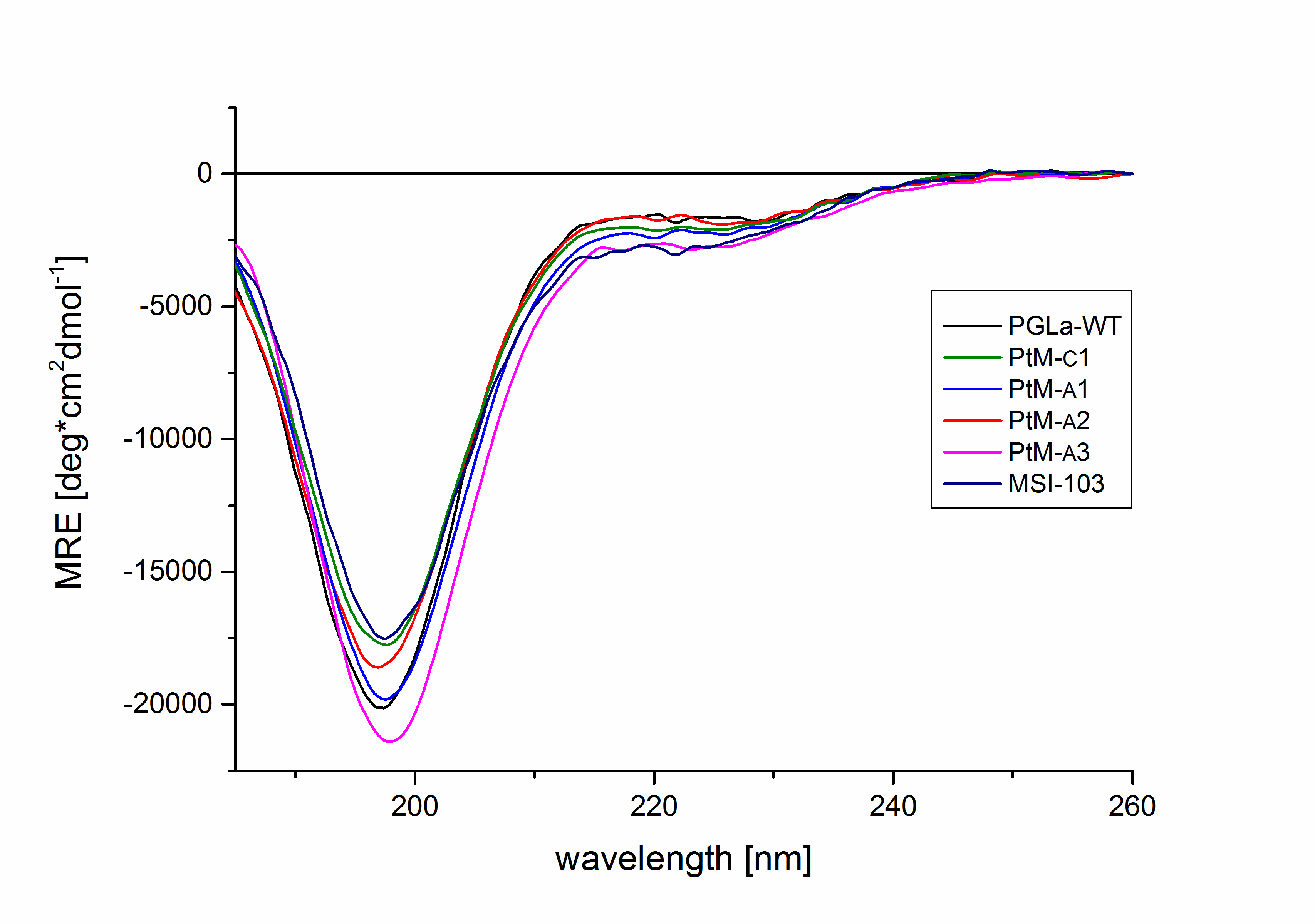 | 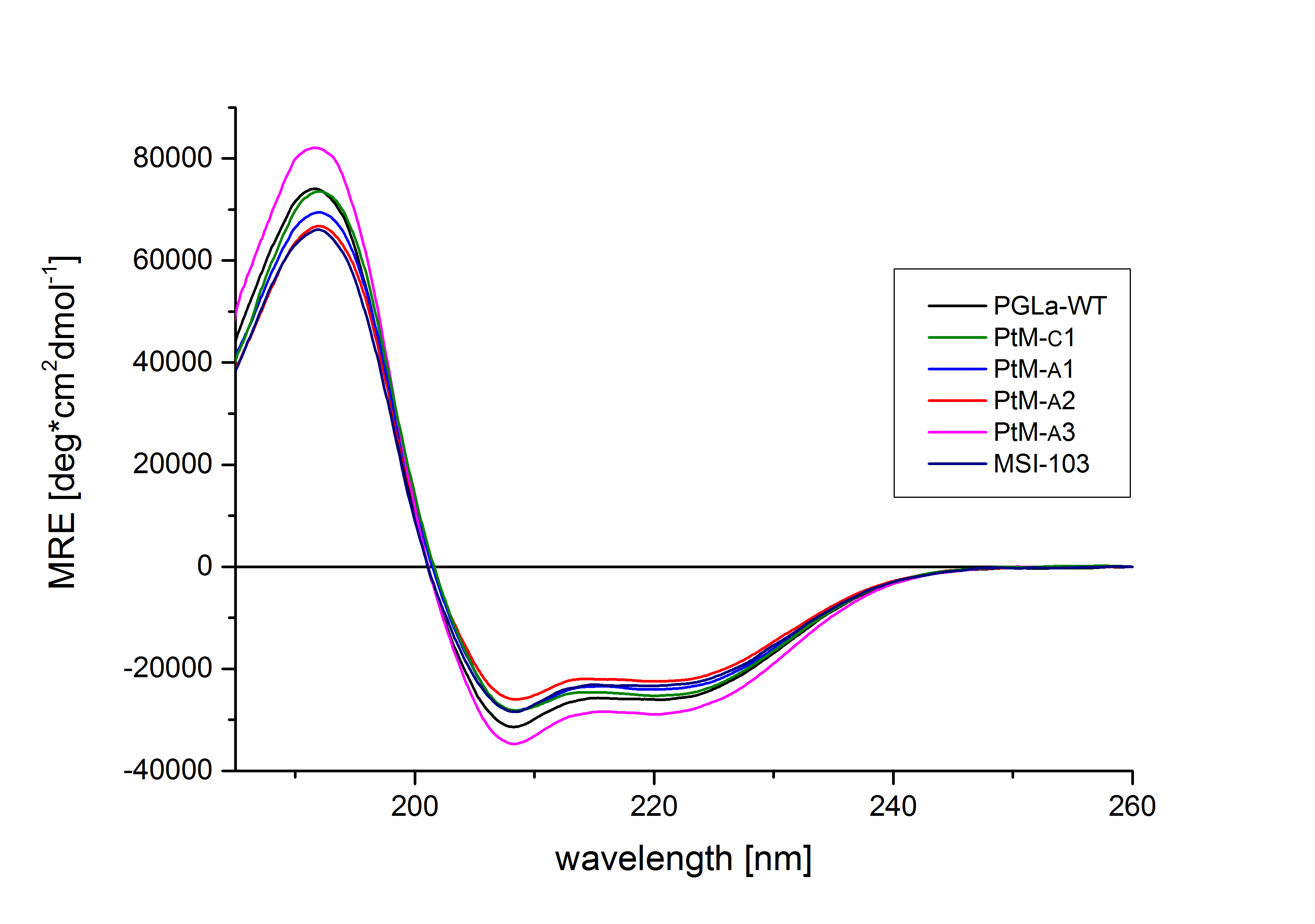 |
| --- | --- |
| (A) | (B) |

**Figure S3. CD spectra of PtM multiple mutants in (A) phosphate buffer and (B) DMPC/DMPG vesicles.** All peptides show spectra typical of random coils in buffer and of α-helices in membranes.

| 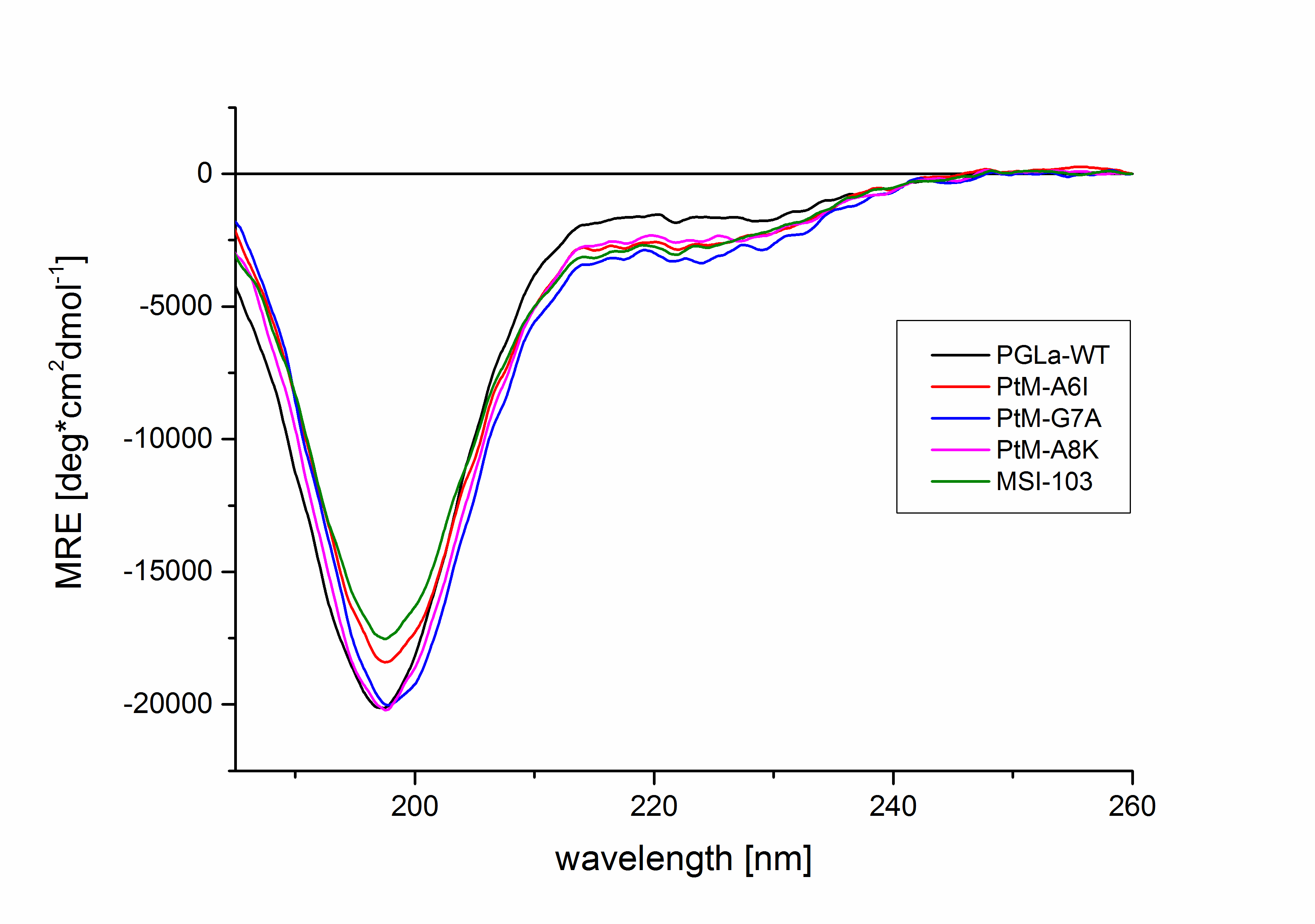 | 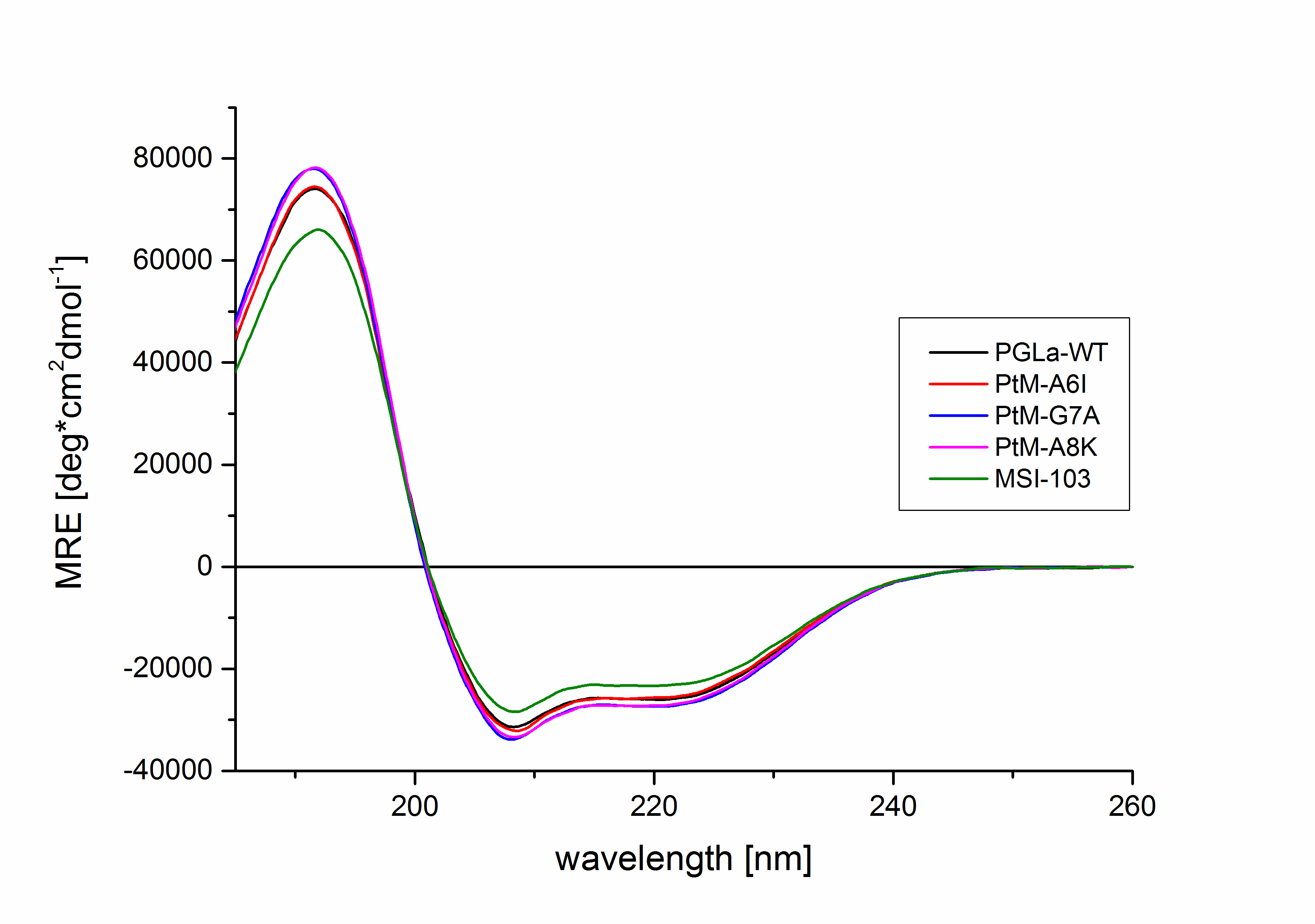 |
| --- | --- |
| (A) | (B) |

**Figure S4. CD spectra of PtM single mutants in (A) phosphate buffer and (B) DMPC/DMPG vesicles.** All peptides show spectra typical of random coils in buffer and of α-helices in membranes.

| 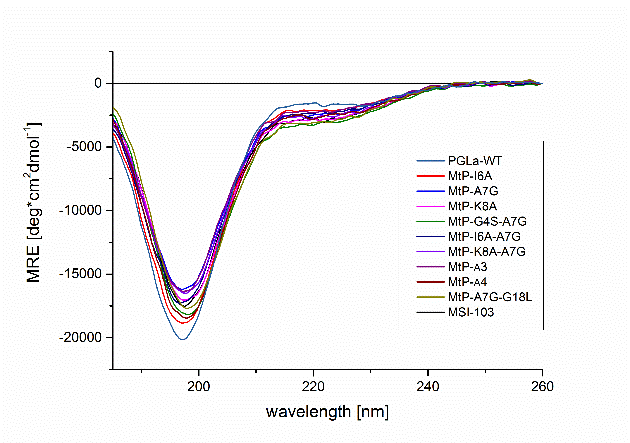 | 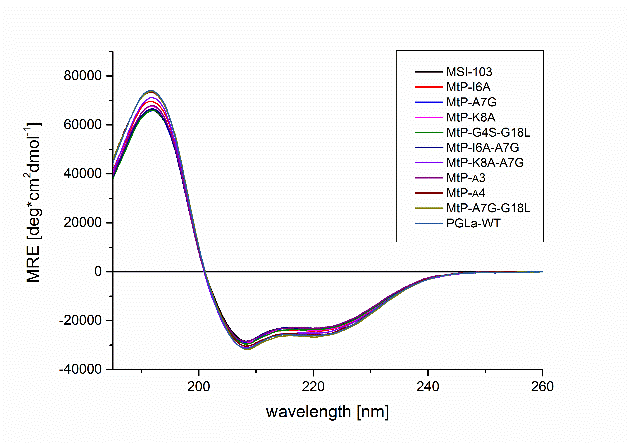 |
| --- | --- |
| (A) | (B) |

**Figure S5. CD spectra of MtP mutants in (A) phosphate buffer and (B) DMPC/DMPG vesicles.** All peptides show spectra typical of random coils in buffer and of α-helices in membranes.

| **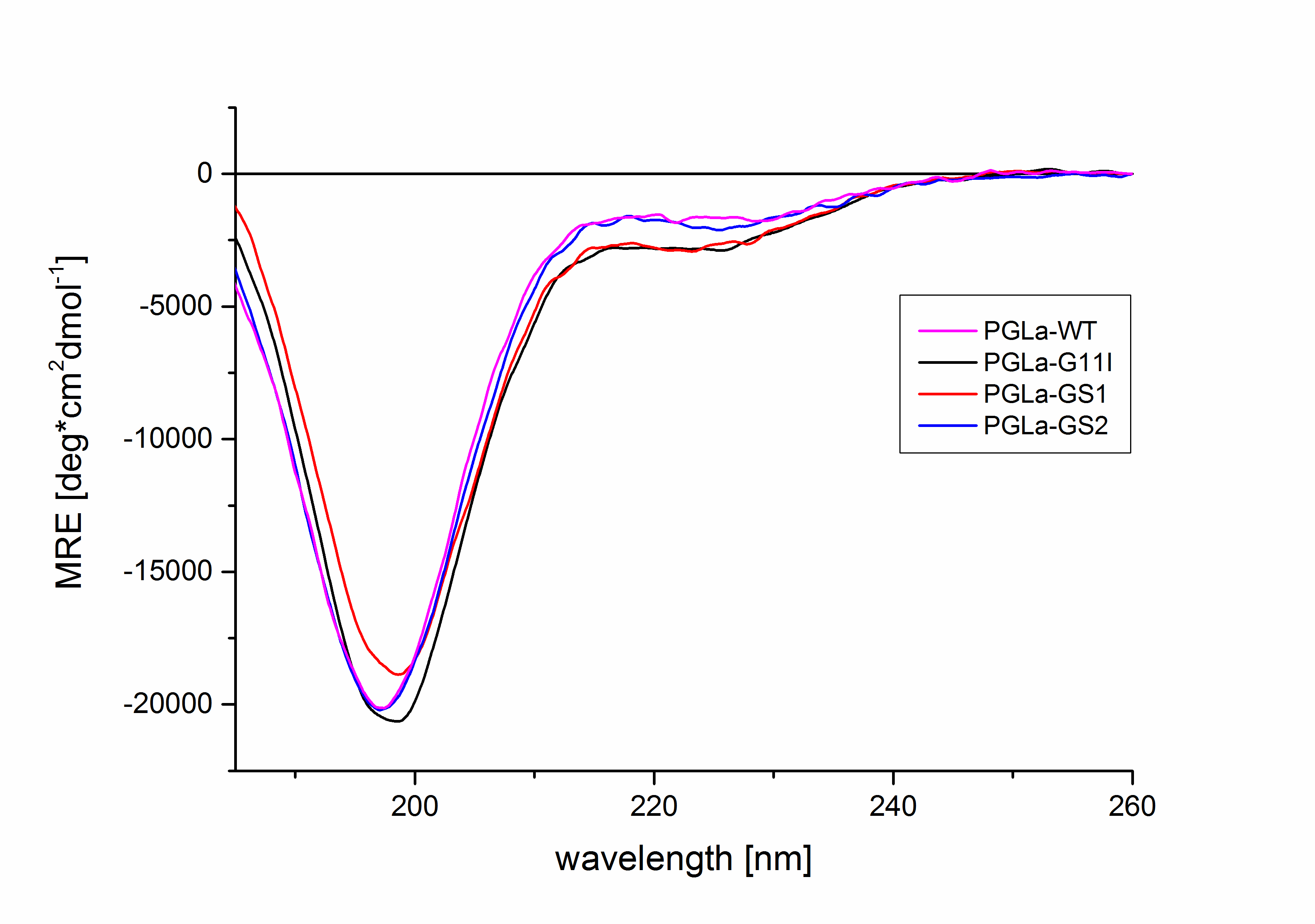** | **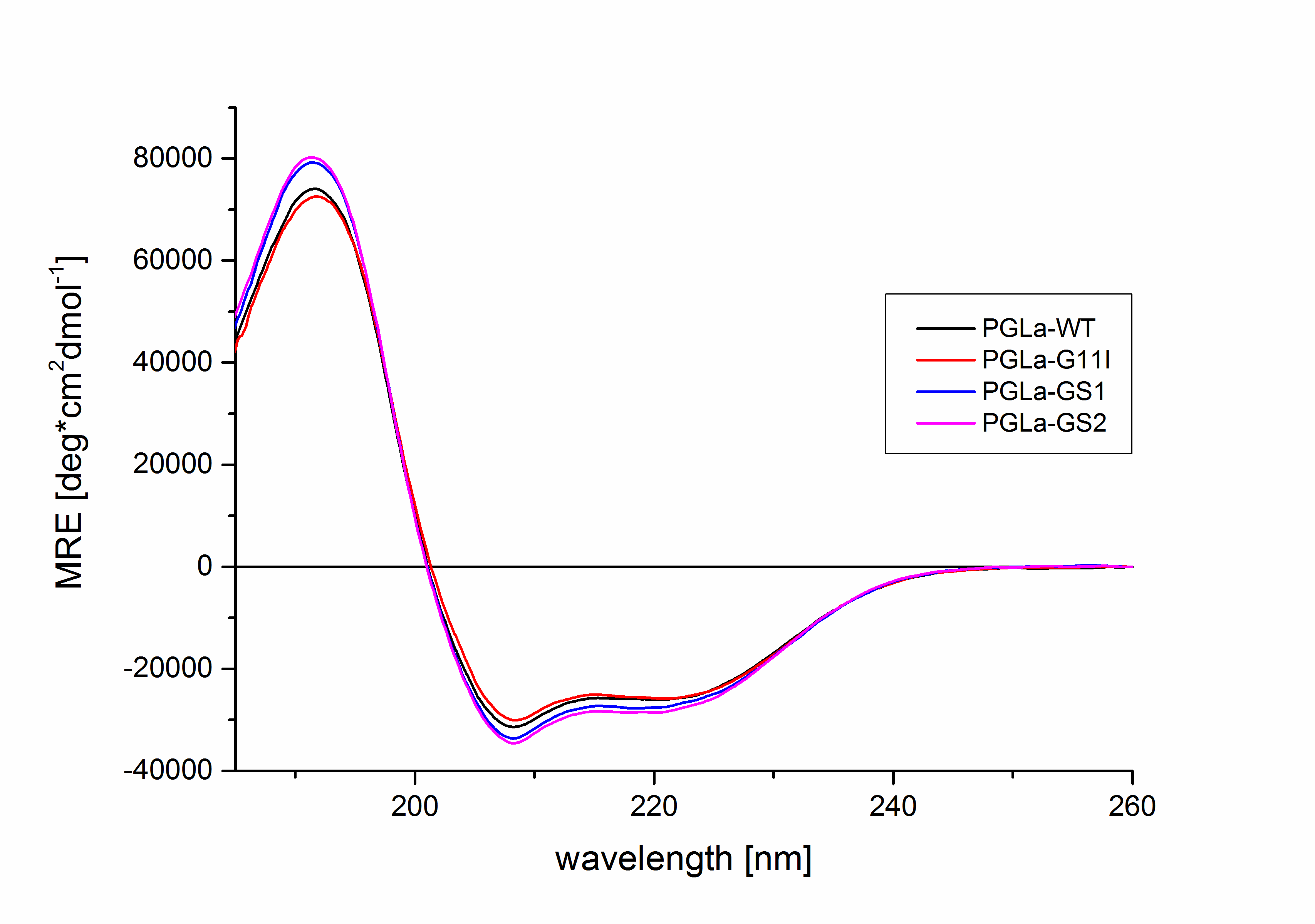** |
| --- | --- |
| (A) | (B) |

**Figure S6. CD spectra of PGLa glycine mutants in (A) phosphate buffer and (B) DMPC/DMPG vesicles.** All peptides show spectra typical of random coils in buffer and of α-helices in membranes.


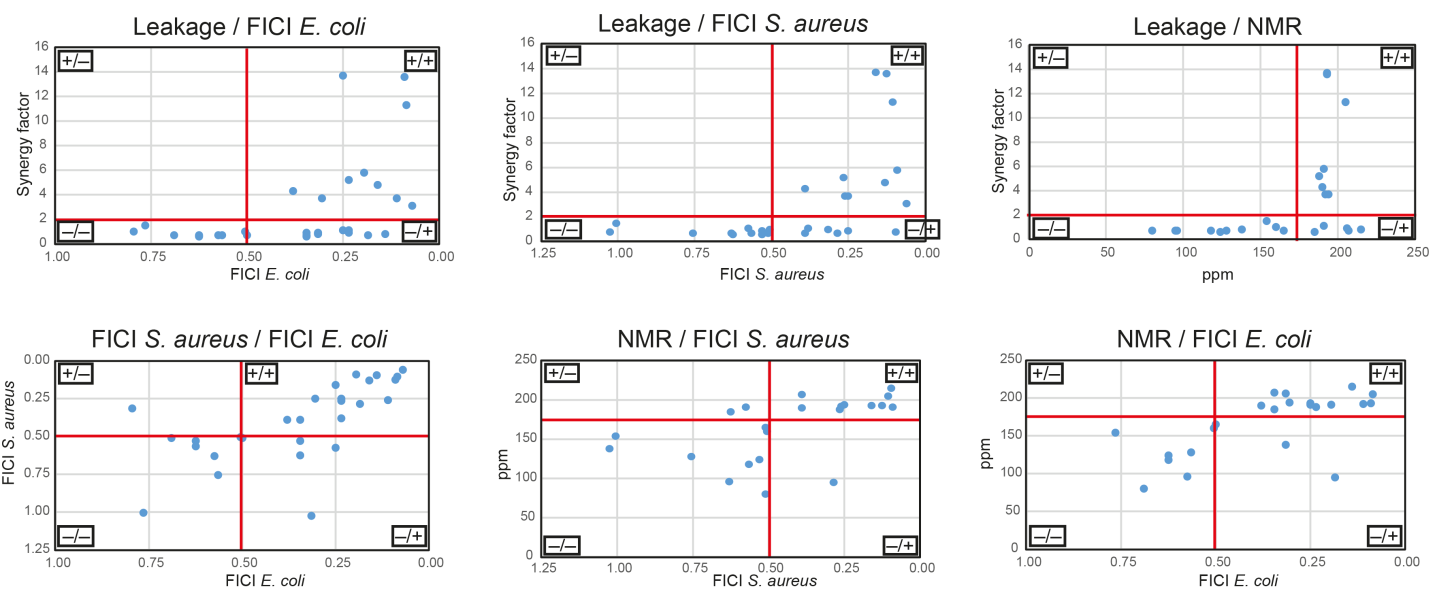


**Figure S7. Correlations between results from the three different methods used to determine synergy.** In the plots, we mark the raw data for each peptide-peptide pair with a blue circle, and mark the border between synergy or no synergy in each case with a red line. In each quadrant of the figure, synergy (+) or no synergy (-) for the two methods being compared are indicated. For leakage, a synergy factor above 2 is taken to indicate synergy. For ^15^N-NMR, a signal above 175 ppm is used as a sign of synergy. Regarding FICI, values below 0.5 indicate synergy. To always show the (+/+) case in the top right quadrant, FICI values are plotted in reversed order.


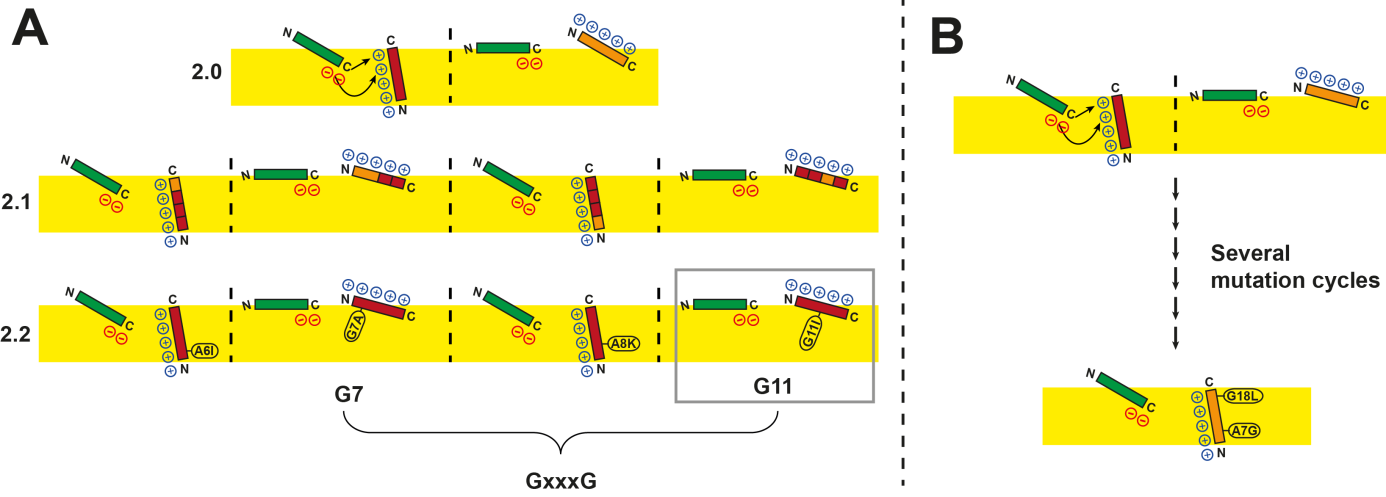
**Figure S8. Comparison of PGLa (red) with MSI-103 (orange) in presence of MAG2 (green).** The orientation of peptides shown was determined from solid-state NMR. (A) As a starting point, mutation cycle 2.0, it was observed that MSI-103 has no synergy with MAG2, and it does not get inserted into the membrane like PGLa in the presence of MAG2. In cycle 2.1, different segments of PGLa were replaced by the corresponding parts of MSI-103, and the region between position 6 and 8 was identified as important for the interaction. In cycle 2.2, single mutations were introduced, showing that Gly7 was a critical residue, whose mutation led to a loss of synergy. The hypothesis that a GxxxG motif could be involved in dimerization was confirmed by cycle 2.3, which showed that Gly11 is also necessary for synergy. (**B**) Mutating MSI-103 (orange) to make it more similar to PGLa (red) and find minimum changes making MSI-103 synergistic with MAG2. Two mutations of MSI-103, A7G and G18L, were enough to make an MSI-103 analogue with full synergy to MAG2.

**SUPPLEMENTARY TABLES**

**Table S1. ^15^**N-NMR chemical shifts of charge mutants.

| Peptide | PGLa-^15^N-A10  [ppm] | PGLa-K19E-^15^N-A10  [ppm] |
| --- | --- | --- |
| + PGLa-^15^N-A10 | 133 | - |
| + PGLa-K19E-^15^N-A10 | - | 165 |
| + MAG2-WT | 205 | 207 |
| + MAG2MAG2-amide | 193 | 205 |
| + MAG2-E19Q | 192 | 197 |
| + MAG2-E19K | 191 | 200 |
| + MAG2-E19K-amide | 185 | 193 |

Table S2. FICIs of charge mutants.

| Peptide combination | *E. coli*  DSM 1103 | | *S. aureus*  DSM 1104 | |
| --- | --- | --- | --- | --- |
|  | 1. FICI | 2. FICI | 1. FICI | 2. FICI |
| PGLa-WT/MAG2-WT | 0.09 | 0.08 | 0.05 | 0.16 |
| PGLa-K5E/MAG2-WT | 0.08 | 0.06 | 0.06 | 0.06 |
| PGLa-K12E/MAG2-WT | 0.13 | 0.19 | 0.13 | 0.13 |
| PGLa-K15E/MAG2-WT | 0.28 | 0.19 | 0.25 | 0.25 |
| PGLa-K19E/MAG2-WT | 0.5 | 0.19 | 0.53 | 0.25 |
| PGLa-WT/MAG2-amide | 0.13 | 0.05 | 0.06 | 0.19 |
| PGLa-WT/MAG2-E19Q | 0.09 | 0.13 | 0.14 | 0.38 |
| PGLa-WT/MAG2-E19K | 0.31 | 0.19 | 0.52 | 0.63 |
| PGLa-WT/MAG2-E19K-amide | 0.38 | 0.31 | 0.5 | 0.75 |

Table S3. Leakage results in POPC/POPG (3:1) vesicles of charge mutants.

| Peptide/Peptide mixture | Leakage [%] | | Synergy factor | |
| --- | --- | --- | --- | --- |
|  | Mean value | Standard deviation | Mean value | Standard deviation |
| PGLa-WT | 2.3 | 0.6 |  |  |
| MAG2-WT | 3.1 | 0.3 |  |  |
| PGLa-WT + MAG2-WT | 60.8 | 3.0 | 11.3 | 1.2 |
| PGLa-K5E | 3.5 | 0.3 |  |  |
| PGLa-K5E + MAG2-WT | 20.7 | 6.6 | 3.1 | 1.1 |
| PGLa-K12E | 3.1 | 0.6 |  |  |
| PGLa-K12E + MAG2-WT | 28.9 | 12.5 | 4.8 | 2.5 |
| PGLa-K15E | 2.9 | 0.3 |  |  |
| PGLa-K15E + MAG2-WT | 5.6 | 2.0 | 0.9 | 0.4 |
| PGLa-K19E | 3.0 | 0.7 |  |  |
| PGLa-K19E + MAG2-WT | 4.1 | 0.1 | 0.7 | 0.1 |
| MAG2-amide | 3.7 | 1.0 |  |  |
| PGLa-WT + MAG2-amide | 79.2 | 4.8 | 13.6 | 2.5 |
| MAG2-E19Q | 5.9 | 1.2 |  |  |
| PGLa-WT + MAG2-E19Q | 30.2 | 5.9 | 3.7 | 0.2 |
| MAG2-E19K | 4.2 | 0.8 |  |  |
| PGLa-WT + MAG2-E19K | 6.8 | 1.2 | 1.1 | 0.2 |
| MAG2-E19K-amide | 2.9 | 0.2 |  |  |
| PGLa-WT + MAG2-E19K-amide | 3.4 | 1.2 | 0.6 | 0.1 |

**Table S4. Leakage results in POPE/POPG/TOCL (72:23:5)** vesicles of charge mutants.

| Peptide/Peptide mixture | Leakage [%] | | Synergy factor | |
| --- | --- | --- | --- | --- |
|  | Mean value | Standard deviation | Mean value | Standard deviation |
| PGLa-WT | 2.2 | 0.8 |  |  |
| MAG2-WT | 1.1 | 0.6 |  |  |
| PGLa-WT + MAG2-WT | 63.5 | 9.1 | 21.4 | 8.4 |
| PGLa-K5E | 2.2 | 1.2 |  |  |
| PGLa-K5E + MAG2-WT | 13.9 | 6.7 | 4.4 | 1.6 |
| PGLa-K12E | 2.3 | 1.0 |  |  |
| PGLa-K12E + MAG2-WT | 40.7 | 5.4 | 12.8 | 3.3 |
| PGLa-K15E | 2.5 | 1.0 |  |  |
| PGLa-K15E + MAG2-WT | 3.5 | 1.7 | 0.9 | 0.2 |
| PGLa-K19E | 1.7 | 0.6 |  |  |
| PGLa-K19E + MAG2-WT | 2.8 | 0.7 | 1.1 | 0.1 |
| MAG2-amide | 0.8 | 1.0 |  |  |
| PGLa-WT + MAG2-amide | 48.3 | 1.6 | 15.2 | 2.7 |
| MAG2-E19Q | 1.1 | 0.4 |  |  |
| PGLa-WT + MAG2-E19Q | 26.6 | 9.9 | 7.5 | 1.9 |
| MAG2-E19K | 1.6 | 0.5 |  |  |
| PGLa-WT + MAG2-E19K | 10.1 | 3.7 | 2.5 | 0.5 |
| MAG2-E19K-amide | 2.9 | 1.0 |  |  |
| PGLa-WT + MAG2-E19K-amide | 7.7 | 1.5 | 1.5 | 0.3 |

**Table S5. ^15^**N-NMR chemical shifts of PGLa-to-MSI mutants.

| Peptide | Chemical shift [ppm] | |
| --- | --- | --- |
|  |  | + MAG2-WT |
| MSI-103-^15^N-A10 | 144 | 128 |
| PtM- c1-^15^N-A10 | 119 | 215 |
| PtM-a1-^15^N-A10 | 152 | 165 |
| PtM-a2-^15^N-A10 | 167 | 191 |
| PtM-a3-^15^N-A10 | 159 | 160 |
| PtM-A6I-^15^N-A10 | 135 | 194 |
| PtM-G7A-^15^N-A10 | 134 | 138 |
| PtM-A8K-^15^N-A10 | 143 | 193 |

Table S6. FICIs of PGLa-to-MSI mutants.

| Peptide combination | *E. coli*  DSM 1103 | | *S. aureus*  DSM 1104 | |
| --- | --- | --- | --- | --- |
|  | 1. FICI | 2. FICI | 1. FICI | 2. FICI |
| MSI-103/MAG2-WT | 0.38 | 0.75 | 1.01 | 0.5 |
| PtM-c1/MAG2-WT | 0.09 | 0.19 | 0.06 | 0.13 |
| PtM-a1/MAG2-WT | 0.5 | 0.5 | 0.5 | 0.52 |
| PtM-a2/MAG2-WT | 0.25 | 0.14 | 0.09 | 0.09 |
| PtM-a3/MAG2-WT | 0.51 | 0.5 | 0.5 | 0.51 |
| PtM-A6I/MAG2-WT | 0.53 | 0.08 | 0.19 | 0.31 |
| PtM-G7A/MAG2-WT | 0.38 | 0.25 | 1.03 | 1.02 |
| PtM-A8K/MAG2-WT | 0.25 | 0.25 | 0.25 | 0.07 |

Table S7. Leakage results in POPC/POPG (3:1) vesicles of PGLa-to-MSI mutants.

| Peptide/Peptide mixture | Leakage [%] | | Synergy factor | |
| --- | --- | --- | --- | --- |
|  | Mean value | Standard deviation | Mean value | Standard deviation |
| PGLa-WT | 2.7 | 0.8 |  |  |
| MAG2-WT | 3.2 | 1.1 |  |  |
| PGLa-WT + MAG2-WT | 60.7 | 2.3 | 11.1 | 4.0 |
| MSI-103 | 2.0 | 0.2 |  |  |
| MSI-103 + MAG2-WT | 3.7 | 0.3 | 0.7 | 0.2 |
| PtM-c1 | 1.4 | 0.4 |  |  |
| PtM-c1 + MAG2-WT | 4.1 | 1.0 | 0.8 | 0.0 |
| PtM-a1 | 4.7 | 1.1 |  |  |
| PtM-a1 + MAG2-WT | 5.5 | 0.5 | 0.7 | 0.0 |
| PtM-a2 | 2.4 | 0.2 |  |  |
| PtM-a2 + MAG2-WT | 27.3 | 4.4 | 5.8 | 0.2 |
| PtM-a3 | 3.3 | 0.4 |  |  |
| PtM-a3 + MAG2-WT | 7.0 | 1.2 | 1.0 | 0.0 |
| PtM-A6I | 2.0 | 0.8 |  |  |
| PtM-A6I + MAG2-WT | 15.6 | 5.9 | 3.7 | 1.6 |
| PtM-G7A | 2.1 | 0.7 |  |  |
| PtM-G7A + MAG2-WT | 4.1 | 0.5 | 0.8 | 0.3 |
| PtM-A8K | 2.5 | 1.1 |  |  |
| PtM-A8K + MAG2-WT | 73.6 | 10.7 | 13.7 | 4.1 |

**Table S8. Leakage results in POPE/POPG/TOCL (72:23:5)** vesicles of PGLa-to-MSI mutants.

| Peptide/Peptide mixture | Leakage [%] | | Synergy factor | |
| --- | --- | --- | --- | --- |
|  | Mean value | Standard deviation | Mean value | Standard deviation |
| PGLa-WT | 2.2 | 0.4 |  |  |
| MAG2-WT | 1.2 | 0.7 |  |  |
| PGLa-WT + MAG2-WT | 60.8 | 1.6 | 19 | 5.2 |
| MSI-103 | 5.6 | 0.8 |  |  |
| MSI-103 + MAG2-WT | 5.7 | 0.9 | 0.9 | 0.3 |
| PtM-c1 | 1.5 | 0.8 |  |  |
| PtM-c1 + MAG2-WT | 3.9 | 1.4 | 1.8 | 0.6 |
| PtM-a1 | 18.6 | 4.0 |  |  |
| PtM-a1 + MAG2-WT | 23.0 | 9.6 | 1.2 | 0.3 |
| PtM-a2 | 4.5 | 1.9 |  |  |
| PtM-a2 + MAG2-WT | 16.1 | 9.6 | 2.9 | 0.4 |
| PtM-a3 | 9.1 | 1.5 |  |  |
| PtM-a3 + MAG2-WT | 13.5 | 3.5 | 1.3 | 0.4 |
| PtM-A6I | 3.7 | 0.7 |  |  |
| PtM-A6I + MAG2-WT | 22.1 | 2.7 | 4.5 | 0.2 |
| PtM-G7A | 3.7 | 0.8 |  |  |
| PtM-G7A + MAG2-WT | 5.8 | 1.8 | 1.1 | 0.2 |
| PtM-A8K | 7.1 | 1.1 |  |  |
| PtM-A8K + MAG2-WT | 60.3 | 7.4 | 7.5 | 2.1 |

**Table S9. ^15^**N-NMR chemical shifts of MSI-to-PGLa mutants.

| Peptide | Chemical shift [ppm] | |
| --- | --- | --- |
|  |  | + MAG2-WT |
| MtP-I6A-^15^N-A10 | 121 | 124 |
| MtP-A7G-^15^N-A10 | 105 | 95 |
| MtP-K8A-^15^N-A10 | 106 | 96 |
| MtP-a3-^15^N-A10 | 125 | 206 |
| MtP-a4-^15^N-A10 | 163 | 188 |
| MtP-A7G-G18L-^15^N-A10 | 159 | 190 |

Table S10. FICIs of MSI-to-PGLa mutants.

| Peptide combination | *E. coli* DSM 1103 | | *S. aureus* DSM 1104 | |
| --- | --- | --- | --- | --- |
|  | 1. FICI | 2. FICI | 1. FICI | 2. FICI |
| MtP-I6A/MAG2-WT | 0.5 | 0.75 | 0.56 | 0.5 |
| MtP-A7G/MAG2-WT | 0.28 | 0.09 | 0.19 | 0.38 |
| MtP-K8A/MAG2-WT | 0.52 | 0.63 | 0.63 | 0.63 |
| MtP-G4S-A7G/MAG2-WT | 0.53 | 1.06 | 0.38 | 0.25 |
| MtP-I6A-A7G/MAG2-WT | 0.16 | 0.31 | 0.38 | 0.38 |
| MtP-K8A-A7G/MAG2-WT | 0.38 | 0.31 | 0.56 | 0.5 |
| MtP-a3/MAG2-WT | 0.38 | 0.25 | a | a |
| MtP-a4/MAG2-WT | 0.28 | 0.19 | 0.28 | 0.25 |
| MtP-A7G-G18L/MAG2-WT | 0.38 | 0.38 | 0.28 | 0.5 |

^a^ MIC not measurable, therefore no FICI determination possible.

Table S11. Leakage results in POPC/POPG (3:1) vesicles of MSI-to-PGLa mutants.

| Peptide/Peptide mixture | Leakage [%] | | Synergy factor | |
| --- | --- | --- | --- | --- |
|  | Mean value | Standard deviation | Mean value | Standard deviation |
| PGLa-WT | 2.3 | 0.6 |  |  |
| MAG2-WT | 3.2 | 0.7 |  |  |
| PGLa-WT + MAG2-WT | 60.3 | 2.2 | 11.8 | 4.0 |
| MtP-I6A | 1.6 | 0.9 |  |  |
| MtP- I6A + MAG2-WT | 3.1 | 1.9 | 0.6 | 0.1 |
| MtP-A7G | 1.4 | 1.1 |  |  |
| MtP- A7G + MAG2-WT | 3.2 | 1.7 | 0.7 | 0.2 |
| MtP-K8A | 1.7 | 0.2 |  |  |
| MtP- K8A + MAG2-WT | 2.3 | 0.6 | 0.7 | 0.2 |
| MtP-G4S-A7G | 1.4 | 0.9 |  |  |
| MtP- G4S-A7G + MAG2-WT | 3.8 | 0.9 | 1.0 | 0.3 |
| MtP-I6A-A7G | 0.8 | 0.7 |  |  |
| MtP- I6A -A7G + MAG2-WT | 3.6 | 1.5 | 1.1 | 0.3 |
| MtP-K8A-A7G | 1.3 | 0.5 |  |  |
| MtP-K8A-A7G + MAG2-WT | 3.2 | 0.8 | 0.9 | 0.0 |
| MtP-a3 | 0.9 | 0.4 |  |  |
| MtP-a3 + MAG2-WT | 3.6 | 0.5 | 0.9 | 0.2 |
| MtP-a4 | 2.5 | 0.1 |  |  |
| MtP-a4 + MAG2-WT | 26.9 | 6.3 | 5.2 | 1.1 |
| MtP-A7G-G18L | 4.9 | 0.3 |  |  |
| MtP- A7G-G18L + MAG2-WT | 34.7 | 3.9 | 4.3 | 0.5 |

**Table S12. Leakage results in POPE/POPG/TOCL (72:23:5)** vesicles of MSI-to-PGLa mutants.

| Peptide/Peptide mixture | Leakage [%] | | Synergy factor | |
| --- | --- | --- | --- | --- |
|  | Mean value | Standard deviation | Mean value | Standard deviation |
| PGLa-WT | 2.1 | 0.8 |  |  |
| MAG2-WT | 1.0 | 0.4 |  |  |
| PGLa-WT + MAG2-WT | 62.6 | 8.1 | 21.9 | 6.7 |
| MtP-I6A | 4.8 | 1.6 |  |  |
| MtP- I6A + MAG2-WT | 4.8 | 1.7 | 0.8 | 0.1 |
| MtP-A7G | 5.1 | 2.0 |  |  |
| MtP- A7G + MAG2-WT | 6.4 | 1.3 | 1.1 | 0.2 |
| MtP-K8A | 1.8 | 0.3 |  |  |
| MtP- K8A + MAG2-WT | 1.9 | 0.5 | 0.8 | 0.3 |
| MtP-a3 | 0.9 | 0.2 |  |  |
| MtP-a3 + MAG2-WT | 2.0 | 0.4 | 1.0 | 0.5 |
| MtP-a4 | 2.6 | 1.1 |  |  |
| MtP-a4 + MAG2-WT | 11.2 | 3.5 | 3.0 | 0.5 |

**Table S13. ^15^**N-NMR chemical shifts of Gly mutants.

| Peptide | Chemical shift [ppm] | |
| --- | --- | --- |
|  |  | + MAG2-WT |
| PGLa-G11I -^15^N-A10 | 143 | 154 |
| PGLa-GS1 -^15^N-A10 | 122 | 118 |
| PGLa-GS2 -^15^N-A10 | 72 | 80 |

Table S14. FICIs of Gly mutants.

| Peptide combination | *E. coli*  DSM 1103 | | *S. aureus*  DSM 1104 | |
| --- | --- | --- | --- | --- |
|  | 1. FICI | 2. FICI | 1. FICI | 2. FICI |
| PGLa-G11I/MAG2-WT | 0.5 | 1.03 | 1.0 | 1.01 |
| PGLa-GS1/MAG2-WT | 0.75 | 0.5 | 0.63 | 0.5 |
| PGLa-GS2/MAG2-WT | 0.75 | 0.63 | 0.51 | 0.51 |

Table S15. Leakage results in POPC/POPG (3:1) vesicles of Gly mutants.

| Peptide/Peptide mixture | Leakage [%] | | Synergy factor | |
| --- | --- | --- | --- | --- |
|  | Mean value | Standard deviation | Mean value | Standard deviation |
| PGLa-WT | 2.2 | 0.7 |  |  |
| MAG2-WT | 3.2 | 0.8 |  |  |
| PGLa-WT + MAG2-WT | 60.4 | 2.3 | 12.3 | 4.8 |
| PGLa-G11I | 11.0 | 5.5 |  |  |
| PGLa-G11I + MAG2-WT | 20.9 | 10.5 | 1.5 | 0.0 |
| PGLa-GS1 | 1.9 | 0.6 |  |  |
| PGLa-GS1 + MAG2-WT | 4.0 | 0.4 | 0.7 | 0.1 |
| PGLa-GS2 | 2.0 | 0.6 |  |  |
| PGLa-GS2 + MAG2-WT | 4.0 | 0.9 | 0.7 | 0.2 |

**Table S16. Leakage results in POPE/POPG/TOCL (72:23:5)** vesicles of Gly mutants.

| Peptide/Peptide mixture | Leakage [%] | | Synergy factor | |
| --- | --- | --- | --- | --- |
|  | Mean value | Standard deviation | Mean value | Standard deviation |
| PGLa-WT | 2.3 | 0.7 |  |  |
| MAG2-WT | 1.1 | 0.6 |  |  |
| PGLa-WT+ MAG2-WT | 60.2 | 2.1 | 18.6 | 4.6 |
| PGLa-G11I | 11.4 | 4.2 |  |  |
| PGLa-G11I+ MAG2-WT | 23.1 | 3.0 | 1.9 | 0.5 |
| PGLa-GS1 | 2.7 | 1.2 |  |  |
| PGLa-GS1 + MAG2-WT | 3.9 | 1.4 | 1.1 | 0.2 |
| PGLa-GS2 | 2.2 | 0.8 |  |  |
| PGLa-GS2 + MAG2-WT | 3.1 | 0.3 | 1.0 | 0.2 |
